# Phosphorylated-tau associates with HSV-1 chromatin and correlates with nuclear speckles decondensation in low-density host chromatin regions

**DOI:** 10.1016/j.nbd.2025.106804

**Published:** 2025-01-14

**Authors:** Leonardo D’Aiuto, Jill K. Caldwell, Terri G. Edwards, Chaoming Zhou, Matthew L. McDonald, Roberto Di Maio, Wood A. Joel, Vanesa R. Hyde, Callen T. Wallace, Simon C. Watkins, Maribeth A. Wesesky, A. Shemesh, Vishwajit L. Nimgaonkar, David C. Bloom

**Affiliations:** a Department of Psychiatry, University of Pittsburgh School of Medicine, Western Psychiatric Institute and Clinic, 3811 O’Hara Street, Pittsburgh, PA 15213, United States of America; b Department of Molecular Genetics & Microbiology, University of Florida College of Medicine, Gainesville, FL 32611, United States of America; c Department of Neurobiology, University of Pittsburgh School of Medicine, 4074 Biomedical Science Tower 3, 3501 Fifth Avenue, Pittsburgh, PA 15213, United States of America; d Department of Neurology, University of Pittsburgh School of Medicine, 3501 Fifth Ave, Biological Science Tower 3, Pittsburgh, PA 15260, United States of America; e Department of Cell Biology, University of Pittsburgh, 3500 Terrace Street, S362 Biomedical Science Tower (South), Pittsburgh, PA 15261, United States of America

**Keywords:** HSV-1, Nuclear speckles, tau phosphorylation

## Abstract

Abnormal tau phosphorylation is a key mechanism in neurodegenerative diseases. Evidence implicates infectious agents, such as Herpes Simplex Virus 1 (HSV-1), as co-factors in the onset or the progression of neurodegenerative diseases, including Alzheimer’s disease. This has led to divergence in the field regarding the contribution of viruses in the etiology of neurodegenerative diseases. Research indicates that viruses may function as risk factors driving neurodegenerative disease rather than playing a causative role. Investigating HSV-1 in abnormal tau phosphorylation is important for understanding the role of infectious agents in neurodegeneration.

We generated cellular models of HSV-1 acute, latent infection, and viral reactivation from latency in cortical brain organoids and investigated the interplay between tau phosphorylation and HSV-1 infection by employing human induced pluripotent stem cell (iPSC)-derived monolayer neuronal cultures and brain organoids. Acute infection with HSV-1 strains 17*syn*^+^ and KOS caused nuclear accumulation of phosphorylated tau (p-tau) in neurons and neural precursor cells. Antivirals prevented nuclear accumulation of p-tau. Viral reactivation was accompanied by the nuclear translocation of p-tau. Chromatin immunoprecipitation analysis indicated an interaction of p-tau with the viral chromatin. A reduction in abundance of component of nuclear speckles and their loss of organized morphology in low-denisty host chromatin regions was observed, with strain-specific differences. HSV-1 infection was followed by an increase in the abundance of BRSKs and TAOKs, kinases known to phosphorylate tau.

These findings show interaction between p-tau and HSV-1 chromatin and demonstrate the ability of HSV-1 to activate mechanisms that are observed in Alzheimer’s disease.

## 1. Introduction

Tau is a microtubule-associated protein responsible for microtubule stabilization (Avila et al., 2004), is primarily concentrated within axons, while its presence in dendrites is notably diminished (Ittner et al., 2010; Kimura et al., 2014), Six tau isoforms are generated by alternative splicing, which exhibit varying level of abundance. Tau protein undergoes post-translational modification, which includes phosphorylation. In the longest tau isoform expressed in the human central nervous system (the 2N4R isoform), there are more than 85 potential phosphorylation sites, which include serine, threonine, and tyrosine residues (Goedert et al., 1989). The interaction of tau with microtubules is mediated by the binding of the positively charged tau proline-rich domain, MTBR, and the negatively charged microtubules (Mukrasch et al., 2007), and it is regulated by site-specific phosphorylation. Phosphorylation at multiple sites, including Ser199, Ser202, Thr205, Ser396, and Ser404, induce conformational changes that lead to misfolding and aggregation of tau protein.

Abnormal tau phosphorylation has been identified as a key pathophysiological mechanism of neurodegenerative diseases, including Alzheimer’s disease (AD), frontotemporal dementia, and progressive supranuclear palsy (Noble et al., 2013). Tau pathology is characterized by the abnormal aggregation of hyperphosphorylated tau protein, linked to neuronal degeneration and death (Di et al., 2016). In addition to genetic and environmental factors, accumulating evidence suggests that infectious agents may contribute to the development and progression of neurodegenerative diseases (Blackhurst and Funk, 2023). In particular, the association between Herpes Simplex Virus 1 (HSV-1) infection and AD has been proposed (Itzhaki et al., 1997; Itzhaki, 2014). HSV-1 has been suggested to be implicated in elevating risk for AD in conjunction with APOE ϵ4 (ApoE4), which represents the major genetic risk factor for sporadic AD (Koller et al., 2020). One copy of the APOE ϵ4 allele increases AD risk approximately 2–3-fold, whereas two APOE ϵ4 alleles increase AD risk approximately 12-fold (Michaelson, 2014; Verghese et al., 2011). HSV-1 has been detected in the brains of AD patients, but it has also been found in the brains of healthy controls (Itzhaki et al., 1997; Jamieson et al., 1991). However, while the incidence of AD is not increased in those with HSV-1 DNA or the APOE4 allele alone, it is highest in the carriers of this allele who also harbor HSV-1 DNA in the CNS (Burgos et al., 2006; Linard et al., 2020). Multiple studies employing monolayer and three-dimensional culture systems and animal models have consistently shown induction of tau phosphorylation at sites that are typically affected in neurodegenerative disorders (DeChiara et al., 2019; Cairns et al., 2020; Powell-Doherty et al., 2020; Alvarez et al., 2012; Wozniak et al., 2009a). It has been postulated that repeated episodes of HSV-1 reactivation from latency in the CNS triggers formation of AD pathology (Itzhaki, 2018). This hypothesis has garnered evidence from a mouse model showing accumulation of proteins relevant to AD pathology including Aβ and phosphorylated tau after repeated cycles of viral reactivation induced by thermal stress (DeChiara et al., 2019). Epidemiological studies have also established an association between anti-herpetic therapy and reduced risk of dementia (Tzeng et al., 2018; Linard et al., 2022; Lopatko Lindman et al., 2021).

However, the mechanism by which HSV-1 contributes to tau pathology in neurodegenerative diseases remains unclear. Thus, investigating the impact of HSV-1 infection on abnormal tau phosphorylation is of great importance and may shed light on the interaction between infectious agents and neurodegeneration.

While tau’s role in microtubule stabilization has been a significant area of study, tau has also been located in the nucleus, where it accounts for approximately 14 % of total tau (Greenwood and Johnson, 1995). Nuclear unphosphorylated tau has been demonstrated to bind DNA, RNA, and chromatin, (Villasante et al., 1981; Wiche et al., 1978; Mansuroglu et al., 2016; Kampers et al., 1999). Nuclear tau has also been found in the nucleolus, where it seems to play a role in the nucleolar organization and/or heterochromatization of rRNA genes (Loomis et al., 1990; Diez and Wegmann, 2020). The binding of tau to DNA, which occurs in a nonspecific manner through its proline-rich domain and the R2 repeats in the microtubule-binding domain (Wei et al., 2008; Qi et al., 2015), depends on its phosphorylation status. Phosphorylation of tau reduces its ability to bind to DNA (Lu et al., 2013). Tau plays a role in regulating the structure of pericentric heterochromatin (PCH), which is crucial for proper gene expression regulation, by binding to satellite DNA sequences in neurons and modulating the PCH structure (Mansuroglu et al., 2016). The loss of tau function has been linked to disruptions in the PCH’s integrity and relaxation of chromatin in neurons in AD patients (Frost et al., 2014). Moderate levels of nuclear tau phosphorylation do not impair its ability to interact with chromatin (Abasi et al., 2024). The progression of Alzheimer’s disease has been associated with alterations in the phosphorylation state of nuclear tau, indicating that the functional impairment of nuclear tau could be a significant pathological event (Abasi et al., 2024; Gil et al., 2020; Hernández-Ortega et al., 2016).

The formation of nuclear tau aggregates can impact RNA processes by changing the composition, dynamics, and organization of nuclear speckles, (membraneless subnuclear structures that play a pivotal role in the regulation of gene expression (Lamond and Spector, 2003)).

In this study, we employed human induced pluripotent stem cell (iPSC)-derived monolayer neuronal cultures and brain organoids to investigate the effects of HSV-1 infection on tau phosphorylation in human brain cortical organoids. Our findings demonstrate that HSV-1 triggers nuclear accumulation of phosphorylated tau in neurons within the developing cortical plate as well as in neural precursor cells (NPCs) in the ventricular/subventricular-like zones of these differentiating cortical structures. Antiviral treatment inhibited nuclear tau accumulation in both HSV-1 strains KOS- and 17*syn*^+^-infected organoids. Importantly, for the first time we provide evidence of a potential interaction of phosphorylated tau with HSV-1 chromatin. Viral reactivation from latency was accompanied by the nuclear accumulation of phosphorylated tau in organoids infected with both HSV-1 strains. Furthermore, we show that nuclear phosphorylation of tau consequent to HSV-1 infection may be independent on the activity of the Cyclin-dependent kinase 5 (Cdk5).

We also show that HSV-1 causes a significant alteration in the abundance of components of nuclear speckles and their loss of organized morphology in the viral replication compartments.

## 2. Results

### 2.1. Tau phosphorylated at sites Ser199/202 and Thr205 co-localize with HSV-1 proteins ICP4 and ICP27 in infected nuclei

#### 2.1.1. Establishment of HSV-1 acute, latency, and reactivation from latency models in cortical organoids

Alvarez et al. have provided compelling evidence regarding the accumulation of phosphorylated tau within the nuclei of productively HSV-1-infected SK-N-MC human neuroblastoma cells (Alvarez et al., 2012). Whether this accumulation occurs during the latency and reactivation phases of the virus is not yet known. Similarly, the significance and the implication(s) of the nuclear accumulation of phosphorylated tau remain unclear.

**Fig. 1.**
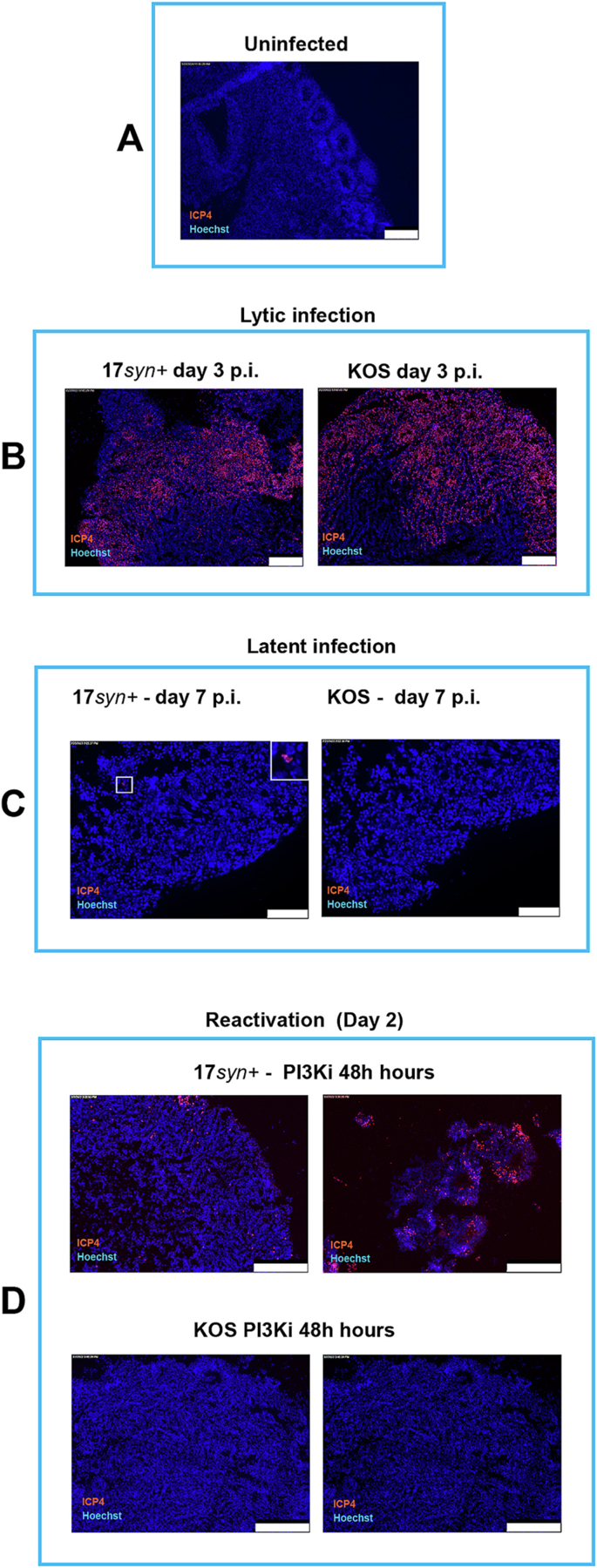
Analysis of the HSV-1 immediate early gene ICP4 expression in HSV-1 acutely, latently infected 51 day-old organoids, and after viral reactivation for latency. (A) Fifty-one-day-old brain organoids were infected individually with HSV-1, strains 17*syn*^+^ and KOS (3000 pfu/organoid). After 2 h the inocula were removed. (B) At day 3 post-infection, organoids were immunostained with ICP4 (red). (C) Under latency conditions, established by culturing infected organoids in the presence of the antivirals (*E*)-5-(2-bromovinyl)-2′-deoxyuridine (5BVdU, 30 μM) and interferon-a (IFN-α, 125 U/ml), ICP4^+^ cells were not detected in KOS-infected organoids. Only a minimal fraction of cells exhibited ICP4 expression in 17*syn*^+^-infected organoids. (D) A substantial increase in ICP4^+^ cells was observed in organoids latently infected with 17*syn* + following 48 h treatment with the PI3 kinase inhibitor LY294002. Conversely, no viral reactivation was detected in those latently infected with KOS. Nuclei were counterstained with Hoechst 33342 (blue). The microphotographs depict peripheral regions of the organoids with the exception of the top-right image in panel D. Scale bars: 250 μm in (A), (B), and (D), and 75 μm in (C).

**Fig. 2.**
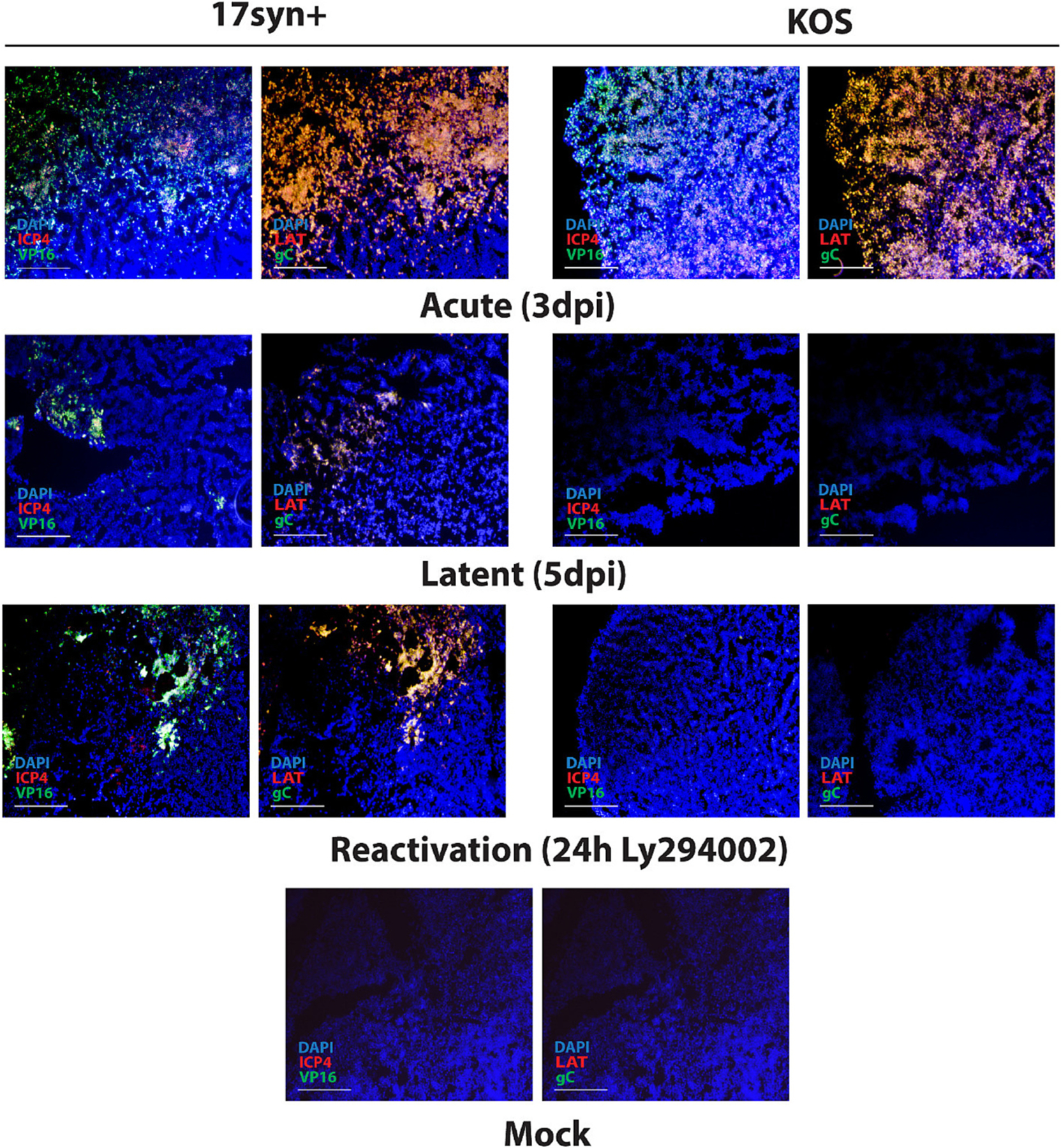
Analysis of HSV-1 transcripts by RNAscope in situ hybridization in acutely and latently infected brain organoids, and after induction of viral reactivation from latency. For each experimental condition, to comprehensively investigate the expression of HSV-1 transcripts, two separate rounds of RNAscope in situ hybridization were performed on the same tissue sections. The first round of RNAscope detected the expression of ICP4 and VP16 transcripts, while the second round targeted LAT and gC. The microphotographs depict peripheral regions of the organoids. Nuclei were counterstained with Hoechst 33342 (blue). Scale bars: 200 μm.

To investigate the first point, we generated cellular models of HSV-1 acute, latent infection, and viral reactivation from latency by employing human cortical brain organoids as follows. Fifty-one-day old cortical organoids (Fig. 1A) were infected with 3000 pfu of HSV-1, as previously described (Abrahamson et al., 2020), using strains 17*syn*^+^ and KOS, as detailed in the methods section. After 2 h, the inocula were removed and organoids were analyzed at day 3 post infection (Figs. 1–2). HSV-1 latency model in cortical organoids was established as previously described (D’Aiuto et al., 2019). Briefly, cortical organoids were pre-treated with antivirals (E)-5-(2-bromovinyl)-2′-deoxyuridine (5BVdU, 30 μM) and interferon-alpha (IFN-α, 125 U/ml) for 24 h (5BVdU + IFN-alpha). The cortical organoids were then infected as described above in the presence of the antivirals 5BVdU + IFN-α. The inocula were removed after two hours and the organoids were cultured in the presence of 5BVdU + IFN-α for 7 days. To induce viral reactivation, latently infected organoids were exposed to phosphatidylinositol 3-kinase inhibitor LY294002 (PI3K, 20 μM) for 48 h.

Immunohistochemistry analysis of frozen sections from acutely infected organoids revealed the presence of cells expressing the HSV-1 immediate early gene ICP4 throughout the organoids, (Fig. 1B). In contrast, no ICP4^+^ cells were detected in KOS-infected organoids in the presence of 5BVdU + IFN-α Figs. 1C and 5A). In the presence of 5BVdU + IFN-α, HSV-1 17*syn*^+^-infected organoids showed minimal levels of ICP4^+^ cells in some areas (Figs. 1C and 5A), consistent with previous findings regarding the difference in suppression of lytic gene expression between strain KOS and 17*syn*^+^ during latency and higher levels of spontaneous reactivation seen with strain 17*syn*^+^ (Grams et al., *2023*). Consistent with these differences, viral reactivation from latency after 48 h treatment of latently-infected organoids with phosphoinositide 3-kinase (PI3K) inhibitor LY294002 was predominantly observed in organoids infected with HSV-1 strain 17*syn*^+^ (Figs. 1D and 5B).

The validity of these 3D models, representing both lytic and latent HSV-1 infections, as well as viral reactivation from latency, was confirmed through RNAscope analysis (Fig. 2). Specifically, RNAscope analysis did not detect cells expressing HSV-1 immediate early, early, and late genes in KOS-infected organoids exposed to antivirals 5BVdU + IFN-α for 5 days, indicating efficient establishment of latency. Conversely, groups of cells expressing the viral genes ICP4, VP16, LAT, and GC were detected in organoids infected with strain17*syn*^+^ in the presence of 5BVdU + IFN-α; however, these groups appeared to be relatively rare (Fig. 2). The differences in relative gene expression during latency seen here between strains 17*syn*^+^ and KOS are consistent with results seen in latent cultures of human LUHMES neurons, with KOS being more transcriptionally silent, but 17*syn*^+^ exhibiting a higher frequency of cells expressing some lytic transcripts, even in the absence of virus production (Grams et al., 2020).

Viral transcripts were detected after 24 h treatment of organoids latently infected with 17*syn*^+^ strain with the PI3K inhibitor LY294002, indicating viral reactivation from latency. No viral transcripts were detected at the same time point in organoids latently infected with strain KOS after treatment with LY294002 (Fig. 2). These results are consistent with the immunohistochemistry data presented in Fig. 1C. It should be noted that we detect little LAT expression in the KOS infected organoids, and this is consistent with a previous study in cultures of differentiated neurons showing that KOS produces less LAT that 17syn^+^ during latency and that this correlates with reduced reactivation (Grams et al., 2020). Also note that since RNAScope probes are strand-specific the LAT probes are outside of the ICP0 coding region so the LAT detected represents the LAT and not ICP0.

#### 2.1.2. Nuclear accumulation of phosphorylated tau in infected organoids in neurons and neural precursor cells

**Fig. 3.**
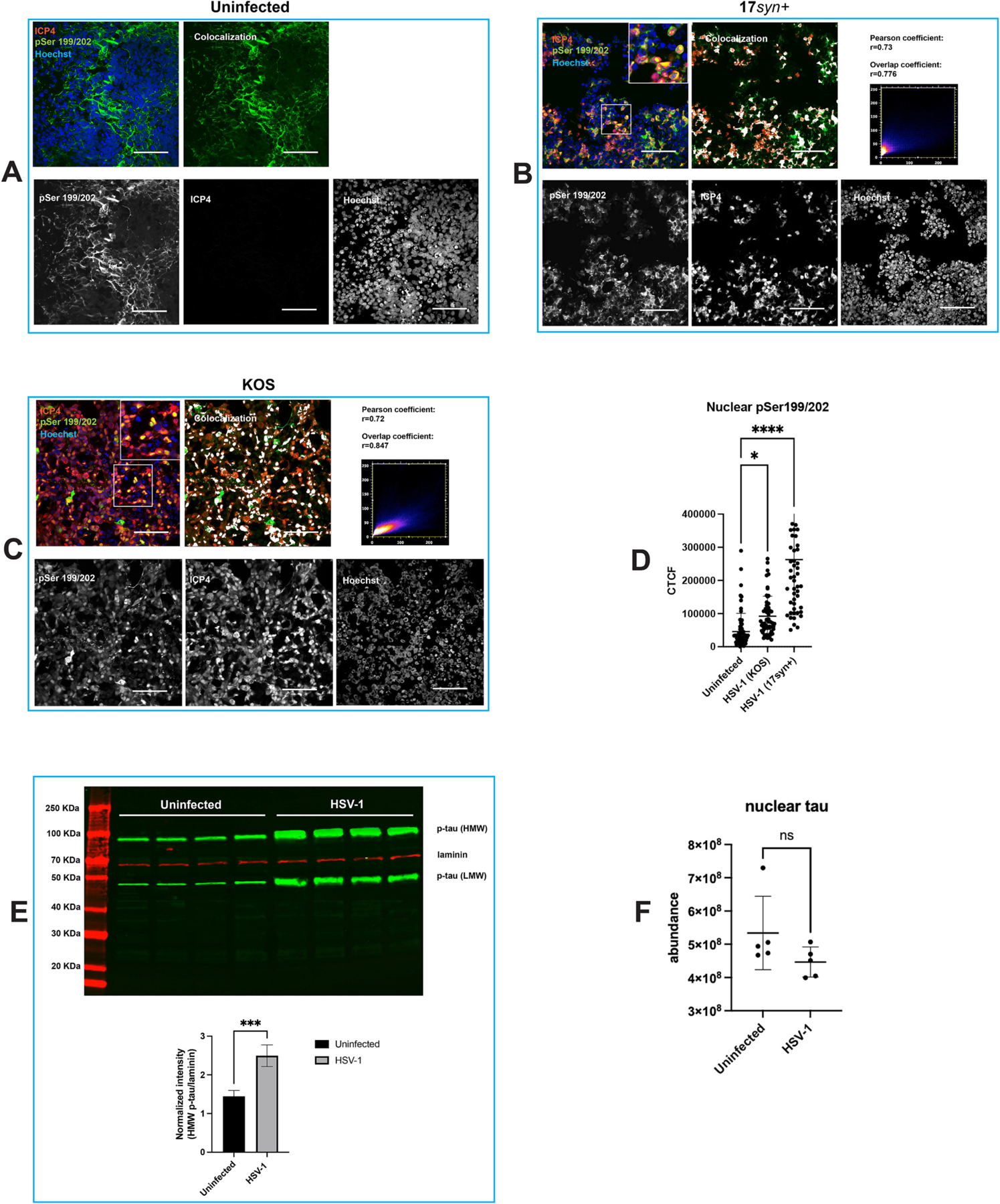
Phosphorylated tau localizes to the nucleus in HSV-1-infected neuronal cells. iPSC-derived cortical brain organoids were infected with HSV-1, strains 17*syn*^+^ and KOS. Infected organoids were processed for immunohistochemistry at day 3 post-infection using phosphorylation-sensitive antibodies targeting ser199/202, as well as an antibody against the HSV-1 protein ICP4. In uninfected organoids tau phosphorylated at Ser199/202 (pSer199/202) (green) was detected only in the cytoplasm (A). Conversely, co-localization of ICP4 (red) and pSer199/202 was observed within the nucleus of 17*syn*^+^*-* and KOS-infected neuronal cells (B-C), indicating a potential interaction between HSV-1 infection and tau phosphorylation. In panels A-C, the colocalization of pSer199/202 and ICP4 was visualized using the Colocalization plug-in feature in Fiji. Quantitative colocalization analysis of pSer199/202 and ICP4 in 17*syn*^+^*-* and KOS- infected organoids is depicted in (B) and (C), respectively. Pearson’s R coefficient and Manders’ overlap coefficient were calculated using JaCoP plug-in feature in Fiji. The fluorescence intensity of nuclear pSer199/202 in uninfected and infected organoids was analyzed by determining the corrected total cell fluorescence (CTCF) (D). Error bars represent standard deviations. One-way analysis of variance (ANOVA) followed by a post-hoc test using the Tukey multiple comparison correction was used to compare the immunofluorescence signals of pSer199/202 between uninfected and infected conditions. *p = 0.0156; ****p < 0.0001. Nuclei were counterstained with Hoechst 33342 (blue). Scale bars: 50 μm. (E) Increase of tau phosphorylated at serine 199/202 in HSV-1 infected neuronal nuclei. Neurons derived from human iPSCs were infected with HSV-1 (strain 17*syn*^+^) at an MOI of 1. Nuclear fractions were isolated from both uninfected cultures and cultures infected for 24 h. The nuclear extracts were then processed for Western blot analysis. (*Top panel)* Immunoblot showing levels of tau phosphorylated at Ser199/202 in uninfected and HSV-1 (stain 17*syn*^+^)-infected iPSC-derived neurons at 24 h post-infection. The blot reveals two distinct bands: a high molecular weight band and a low molecular weight band. The high molecular weight band likely represents hyperphosphorylated tau, while the low molecular weight band may indicate normal or less phosphorylated tau species. (*Bottom panel*) Normalized phosphorylated tau (p-tau) expression. Normalized data are presented as the ratio of p-tau to lamin signal intensity. The experiment was conducted in quadruplicate. Error bars represent standard deviations. Statistical significance was determined using an unpaired Student’s *t*-test, with *p* = 0.0006 (***). (F) HSV-1 infection causes a non-significant change in the abundance of total nuclear tau in HSV-1-infected cultures. Mass spectrometry analysis was performed on nuclei isolated from both uninfected iPSC-derived neuronal monolayer cultures and iPSC-derived neuronal monolayer cultures infected with HSV-1 (strain 17*syn*^+^) at a multiplicity of infection (MOI) of 1 for 24 h, revealing a nonsignificant change in the abundance of nuclear unphosphorylated tau in HSV-1-infected cultures. The experiments were performed in quintuplicate. Error bars represent standard deviations. *P* value was determined using Student’s t-test.

**Fig. 4.**
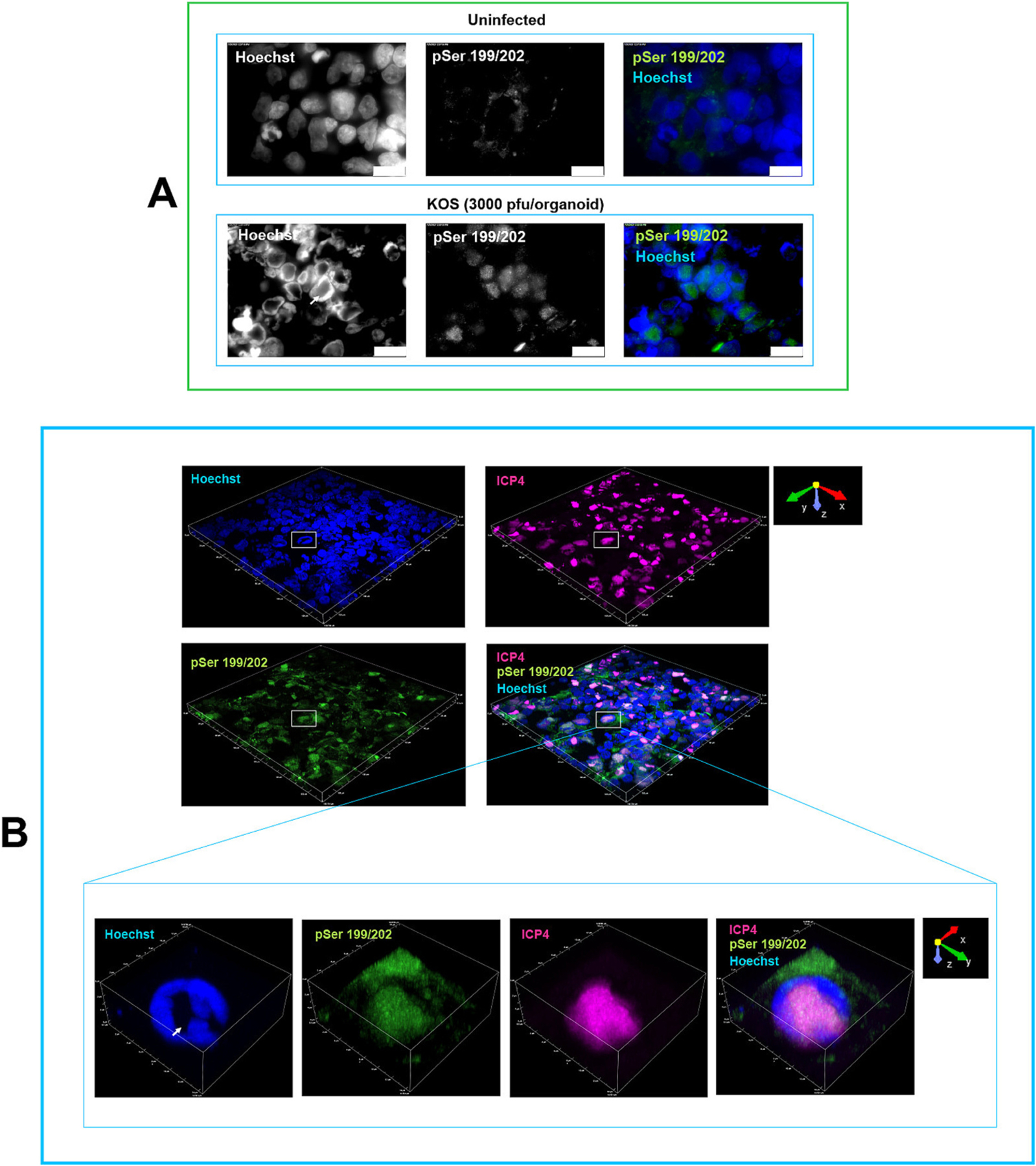
Phosphorylated tau accumulates in low-density host chromatin regions in HSV-1-infected cells. Monolayer cultures of iPSC-derived neurons were infected with HSV-1 at a multiplicity of infection (MOI) of 1, while brain organoids were infected with 3000 plaque-forming units (PFU) per organoid. (A) Infected monolayer cultures where immunostained for pSer199/202 at 24 h post-infection. Nuclei were counterstained with Hoechst 33342. The immunofluorescence signals and Hoechst staining are presented with separate channels displayed in grayscale. Phosphorylated tau accumulated in Hoechst-negative regions. (B) Infected brain organoids where immunostained for pSer199/202 (green) and ICP4 (red) at day 3 post-infection. 3D confocal z-stack imaging of HSV-1 infected brain organoids revealed phosphorylated tau (green) accumulating in low-density host chromatin regions in infected nuclei and colocalizing with ICP4. Nuclei were counterstained with Hoechst 33342 (blue). A low-density host chromatin region is indicated by an arrow. Scale bars:10 μm in top panel.

We sought to investigate the impact of HSV-1 infection on brain organoids by performing immunofluorescence analysis using phosphorylation-sensitive antibodies targeting Ser199/202, Thr205, and Ser396 (these sites were selected because of their significance in neurodegenerative diseases (Noble et al., 2013; Neddens et al., 2018; Wegmann et al., 2021)), as well as antibodies against the HSV-1 proteins ICP4 and ICP27, two viral proteins critical for the replication of herpes simplex virus (Rivas et al., 2021) (Figs. 3–4 and Supplementary Figs. 1–3).

Immunohistochemistry analysis of uninfected cortical organoids showed that tau phosphorylated at pSer199/202 exhibited a cytoplasmic localization (Fig. 3A). In HSV-1-infected organoids, our analysis revealed an accumulation of phosphorylated tau in Hoechst-negative regions of a subset of nuclei infected with HSV-1, as detected by the antibody targeting phosphorylated tau at the Ser199/202 sites. The staining pattern exhibited distinctive foci within the nuclei, which colocalized with ICP4 (Figs. 3B–C, and Figs. 4A–B) and ICP27 (Supplementary Fig. 2) in Hoechst negative regions, indicative of nuclear areas with low host chromatin density formed by displacement of host chromatin during viral infection. These Hoechst negative regions are associated with viral replication compartments, which represent sites of viral replication, viral gene expression and packaging (Seyffert et al., 2021). HSV-1 transcription factor ICP4 can be considered a proxy for HSV-1 viral replication compartments (Seyffert et al., 2021). The quantification of tau phosphorylation levels analyzed by measuring pSer199/202 fluorescence intensity showed a significant increase of phosphorylated tau in the nuclei of KOS- and 17*syn*^+^-infected organoids (Fig. 3D). A quantitative colocalization analysis of pSer199/202 and ICP4 in HSV-1 infected organoids is shown in Figs. 3B–C. These data are in line with the results of Alvarez et al. (Alvarez et al., 2012), who reported nuclear accumulation and colocation of hyperphosphorylated tau and ICP4 in HSV-1 infected SK-N-MC human neuroblastoma (Alvarez et al., 2012). Furthermore, a significant increase of tau phosphorylated at Ser199/202 (*p* = 0.0006) was detected in the nuclear fractions of iPSC-derived monolayer cultures of neurons infected with HSV-1 (strain 17*syn*^+^; MOI of 1) at 24 hpi (Fig. 3E). A non-significant reduction of total nuclear tau was detected in infected monolayer neuronal cultures using proteomic analysis (Fig. 3F). This outcome aligns with our recent findings of non-significant changes in total tau levels in HSV-1-infected neurons (Hyde et al., 2024). These results suggest that the observed increase in nuclear phosphorylated tau is due to post-translational modification rather than an overall increase in tau protein.

Time course analysis of acutely infected organoids (strain KOS) showed that accumulation of tau phosphorylated at Ser199/202 and its co-localization with the ICP4 was evident at 24 h post-infection (Supplementary Fig. 1).

Next, we examined the localization of tau phosphorylated at Thr205 and Ser396 in uninfected and infected organoids (Supplementary Fig. 3). In uninfected organoids, tau phosphorylated at Thr205 and Ser396 exhibited cytoplasmic localization (Supplementary Figs. 3 A and 3D). Conversely, we observed nuclear accumulation of tau phosphorylated at Thr205 and its co-localization with ICP4 within the infected nuclei, (Supplementary Figs. 3B–C). However, we did not observe clear and unambiguous detection of tau phosphorylated at Ser396 in infected nuclei (Supplementary Figs. 3E–F).

**Fig. 5.**
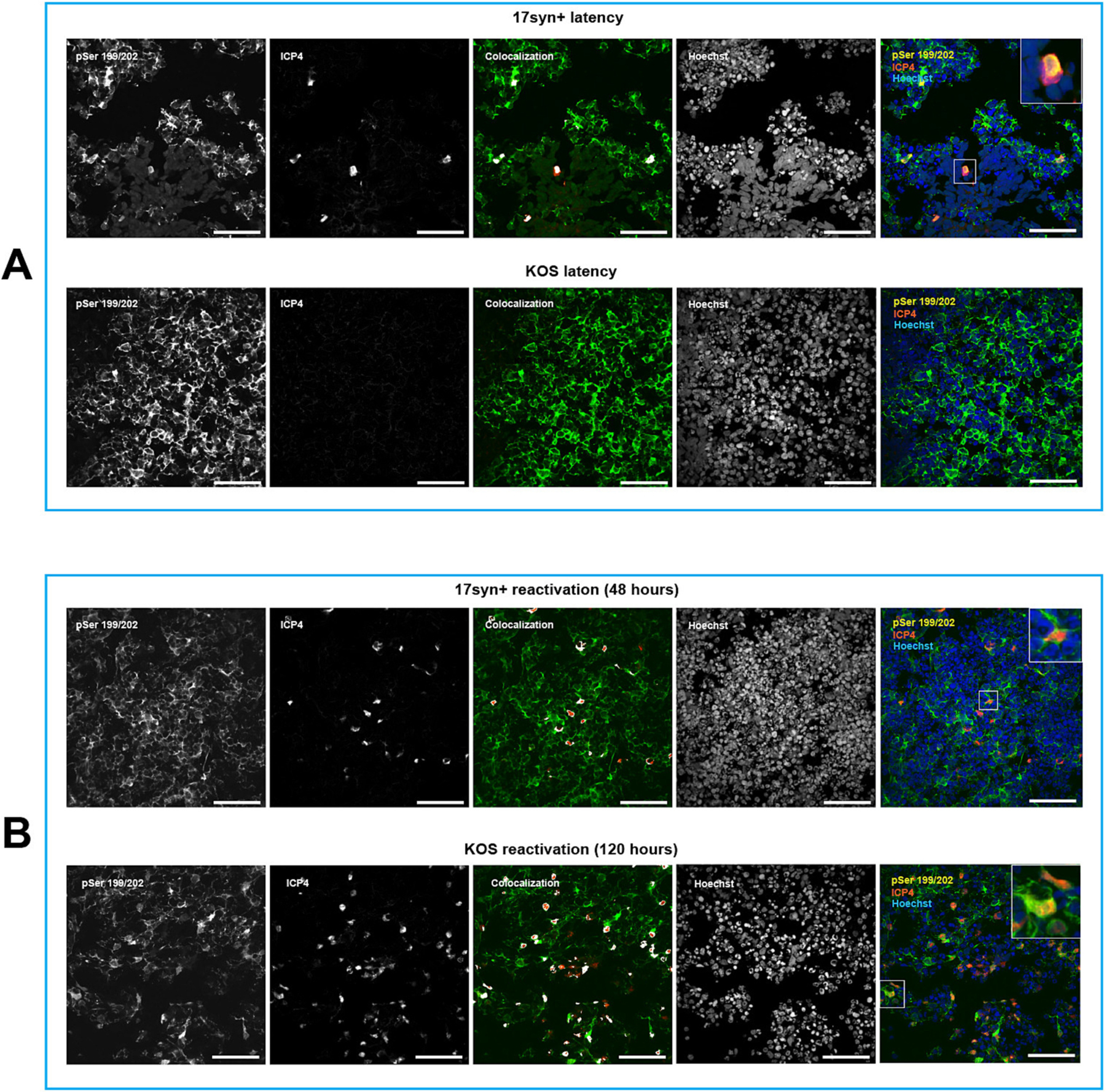
HSV-1 reactivation from latency is followed by nuclear accumulation of phosphorylated tau. Model of HSV-1 latency and reactivation was established in cortical brain organoids using antivirals (E)-5-(2-bromovinyl)-2′-deoxyuridine and interferon-a (A). Viral reactivation from latency was induced by treatment with phosphatidylinositol 3-kinase inhibitor LY294002 (B). Infected cultures in latency conditions and after induction of viral reactivation where immunostained for phosphorylated tau (pSer199/202) and the viral protein ICP4. Organoids infected with 17*syn* + in the presence of antivirals showed the presence of sporadic ICP4-positive cells, indicating that either occasional cells exhibit leaky immediate early gene expression even in the presence of antiviral treatment, or that the establishment of latency in some cells is delayed or inefficient (A, *Top panel*). In contrast, organoids infected with KOS did not exhibit any ICP4-positive cells, consistent with the observation that KOS genomes are more transcriptional repressed in organoid neurons, mirroring observations comparing KOS and 17*syn* + in other in vitro and in vivo models. (A, *Bottom panel*). The nuclear accumulation of phosphorylated tau was detected in ICP4-positive cells upon viral reactivation in 17*syn* + -infected organoids (after 48 h treatment with LY294002) (B, *Top panel*) as well as in KOS-latently infected organoids (after 120 h treatment with LY294002) (B, *Bottom panel*), suggesting that viral-induced mechanisms may contribute to alteration in tau phosphorylation levels and subcellular localization within affected neurons. Nuclei were counterstained with Hoechst 33342 (blue). Scale bars: 50 μm.

Subsequently, we focused on pSer199/202 to investigate a facet of tau phosphorylation in the context of viral infection. No nuclear localization of phosphorylated tau was observed in KOS-infected cultures exposed to antivirals 5BVdU + IFN-α (Fig. 5A), suggesting that the nuclear accumulation of phosphorylated tau is a host response to the viral replication. In organoids infected with HSV-1 strain 17*syn*^+^ under antiviral treatment, cells expressing HSV-1 genes were detected at a low frequency (Fig. 5A). In these cells, nuclear co-localization of ICP4 and Ser199/202 was detected (Fig. 5A and Supplementary Fig. 4 A). These results provide further evidence supporting the relationship between the nuclear accumulation of phosphorylated tau in low-density host chromatin regions and the expression of HSV-1 genes.

After a 48-h treatment with the PI3K inhibitor LY294002, viral reactivation from latency was observed in organoids that were latently infected with 17*syn*^+^ (Fig. 1D, Fig. 2, and Fig. 5B). The presence of nuclear phosphorylated tau was detected, co-localizing with the viral protein ICP4 (Fig. 5B and Supplementary Fig. 4B). Nuclear phosphorylated tau was discernible in ICP4^+^ cells in localized areas of organoids latently infected with KOS after 120 h of exposure to LY294002 (Fig. 5B). The difference between HSV-1 strains KOS and 17*syn*^+^ to establish latency and reactivation evidenced in this study is in agreement with a recent report by Grams et al. (Grams et al., 2020)

**Fig. 6.**
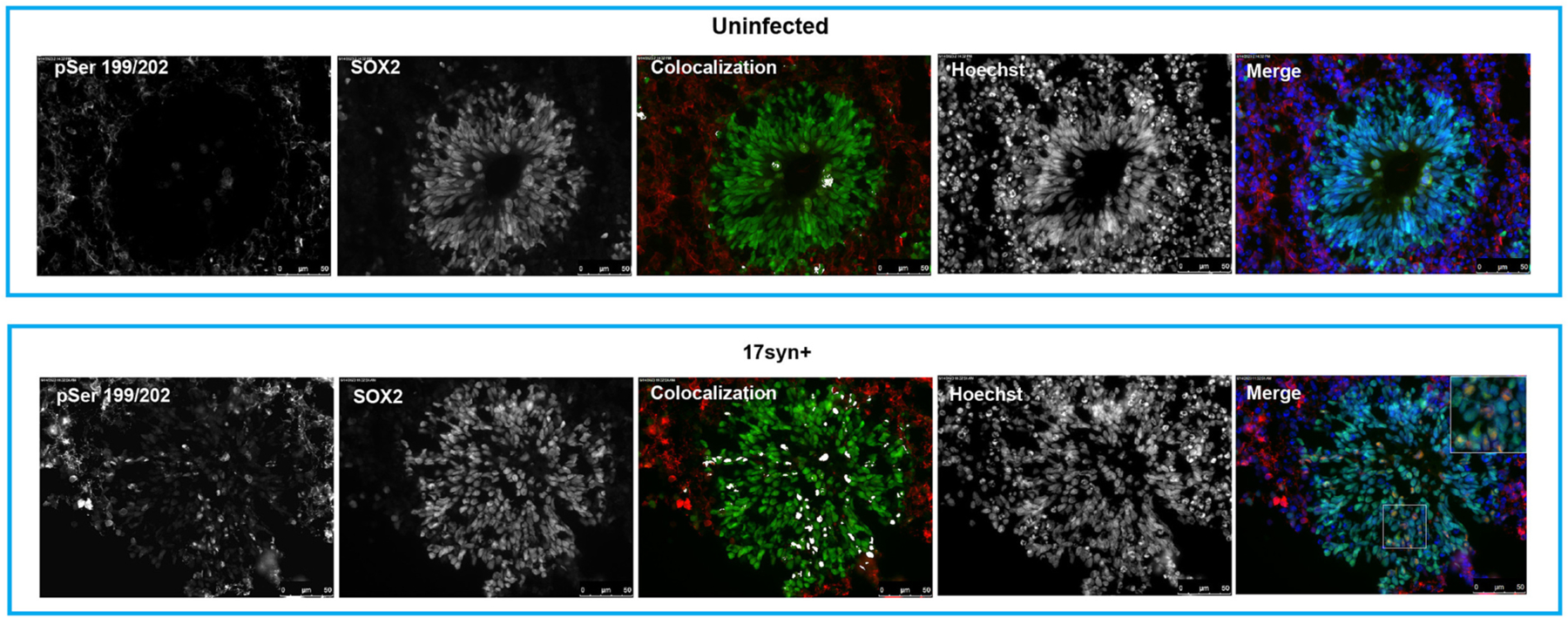
Nuclear accumulation of phosphorylated tau is not limited to neuronal cells, but it also occurs in neural precursor cells (NPCs) in ventricular/subventricular zone region in infected organoids. Cortical organoids were infected with HSV-1, strain 17*syn*^+^, and analyzed at day 3 post-infection. Co-immunostaining for the NPC marker SOX2 and pSer199/202 provided evidence of nuclear accumulation of phosphorylated tau in infected NPCs. The immunofluorescence signals are presented with separate channels displayed in grayscale. Nuclei were counterstained with Hoechst 33342 (blue). The colocalization of pSer199/202 with SOX2 was visualized using the Colocalization plug-in feature in Fiji. Scale bars: 50 μm.

Cells exhibiting nuclear accumulation of phosphorylated tau were also detected in the ventricular/subventricular zone-like regions, which are predominantly composed of NPCs (SOX2^+^) (Qian et al., 2016) (Fig. 6). Phosphorylated tau is not typically detected under normal conditions (Fig. 6, *Top panel*). Co-immunostaining of 17*syn*^+^-infected organoids (day 3 post-infection) for pSer199/202 and the NPC marker SOX2 provided evidence of nuclear accumulation of phosphorylated tau in NPCs following HSV-1 infection (Fig. 6, *Bottom panel*). This finding indicates that nuclear accumulation of phosphorylated tau is not restricted to HSV-1-infected neurons but also occurs in infected NPCs that represent earlier stages during neuronal differentiation.

Combined, our results provide further evidence supporting the relationship between the nuclear accumulation of phosphorylated tau and the expression of viral genes and/or viral replication, indicating a potential link between tau phosphorylation and the host response to viral infection. These findings indicate the possibility of an interaction between phosphorylated tau and viral components.

### 2.2. Phosphorylated tau interacts with HSV-1 chromatin

**Fig. 7.**
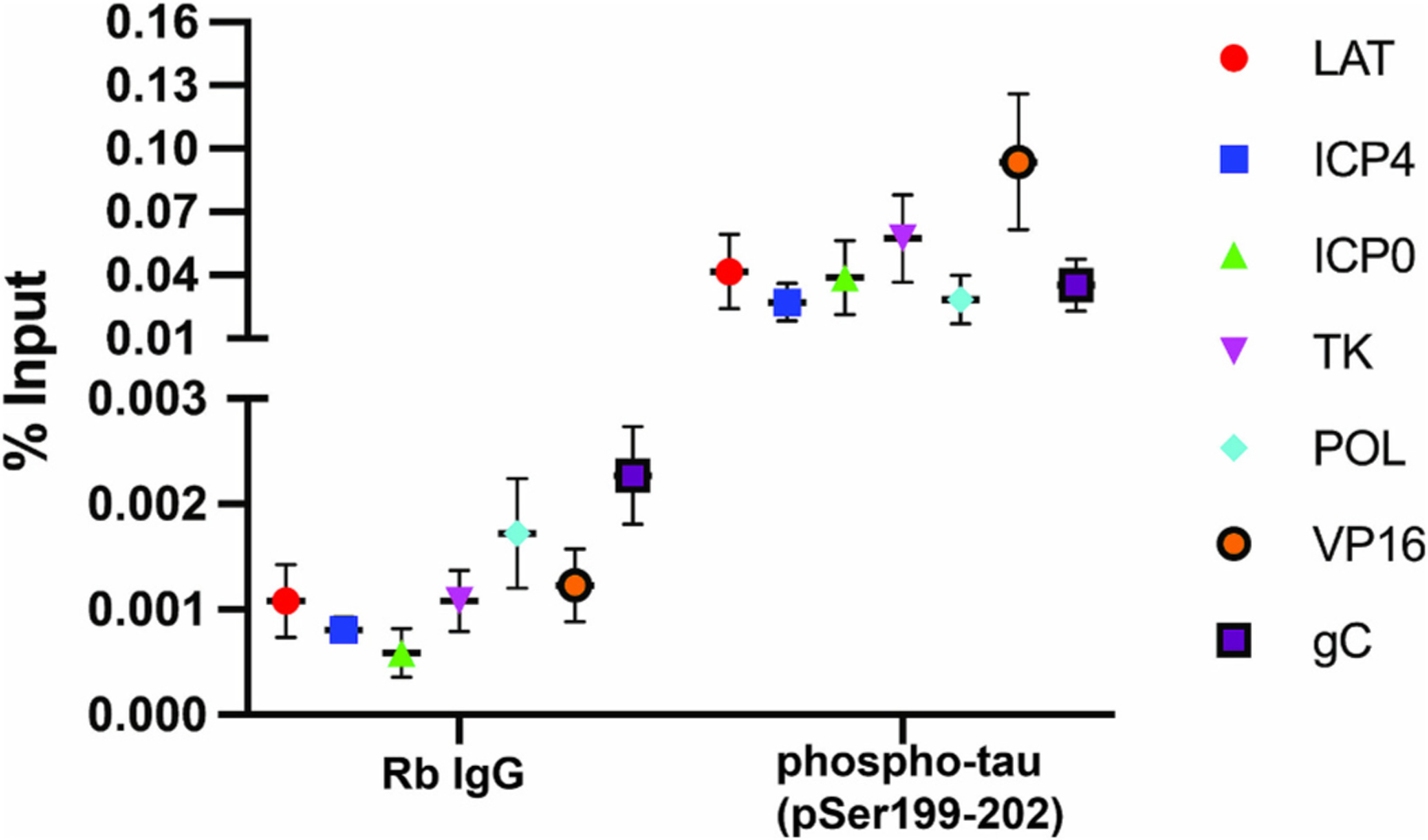
Phosphorylated tau interacts with HSV-1. To investigate the potential interaction between phosphorylated tau and HSV-1 chromatin, HSV-1-infected iPSC-derived monolayer neuronal cultures were analyzed by ChIP-qPCR. An antibody raised against Ser 199/202 of tau along with an isotype control antibody were used for ChIP. qPCR was conducted across seven viral gene regions and data analyzed using the percent input method (see equation in Methods Section). The data represent three independent ChIPs. A significant interaction was found between HSV-1 chromatin and phosphorylated tau compared to the control antibody ChIPs (Unpaired *t*-test, two-tailed: *n* = 21, *p* ≤0.0001, *t* = 4.647, df = 40).

To investigate whether an interaction occurs between phosphorylated tau and HSV-1 chromatin, we employed chromatin immunoprecipitation (ChIP) analysis on uninfected and infected iPSC-derived neuronal cultures. The reason for employing two iPSC-derived culture systems stems from the fact that the distribution of infected areas and the extent of the infection in brain organoids vary not only among different organoids but also among different sections of the organoids. While several outer cell layers are consistently infected at day 3 post infection, the extent of the infection from the periphery to the center varies varied among different organoids. Thus, to ensure more uniform infections for ChIP analysis, we preferred to use monolayer cultures of iPSC-derived neurons. ChIP-QPCR analysis was performed using anti-phospho-tau pSer199/202 antibody. Our results revealed a significant enrichment of phosphorylated tau on HSV-1 chromatin at all regions analyzed, compared with rabbit IgG ChIP. The highest relative enrichment seen at the region encoding VP16, and the least seen at the region encoding ICP4 (Fig. 7).

These results indicate that phosphorylated tau associates with the HSV-1 chromatin in the nucleus and suggests a potential interaction between tau and viral components. This result warrants further investigation to determine if tau phosphorylation might influence HSV-1 gene expression or aspects of HSV-1 life cycle.

#### 2.2.1. HSV-1 downregulation of p35 activator of Cdk5 in iPSC-derived neurons

**Fig. 8.**
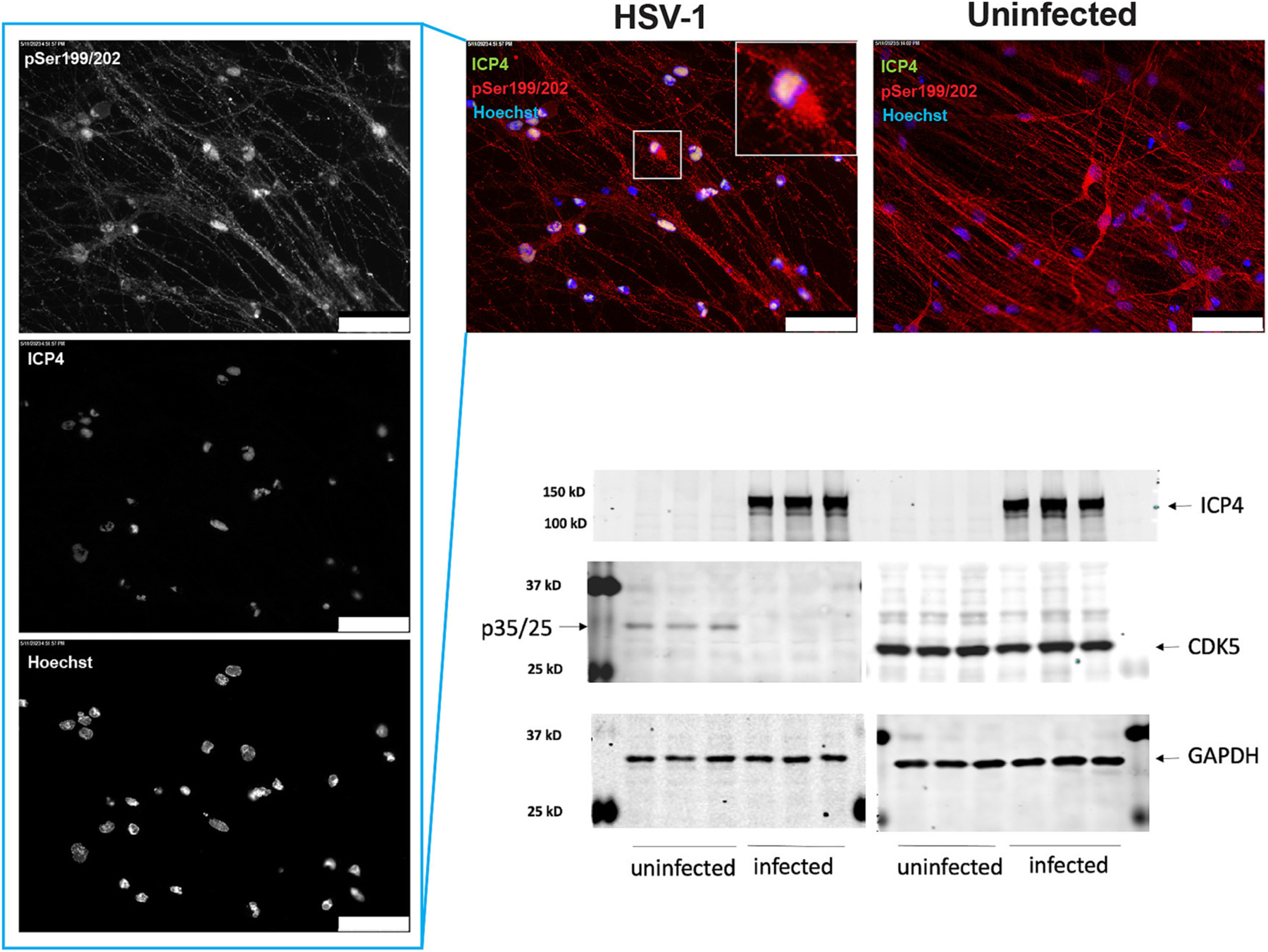
HSV-1 infection leads to downregulation of the p35 activator of Cyclin-dependent kinase 5 (Cdk5) in iPSC-derived neurons. Monolayer cultures of iPSC-derived neurons were infected with HSV-1 at a multiplicity of infection (MOI) of 1. Immunocytochemistry analysis showed nuclear accumulation of phosphorylated tau (pSer199/202) (red) and its colocalization with the viral protein ICP4 (green) at 24 h post-infection. At this time point, aberrant activation of Cdk5, resulting from the cleavage of p35 to p25 mediated by calpain, which leads to tau hyperphosphorylation, was investigated. Western blotting revealed a significant downregulation of p35 with no presence of p25. This finding does not substantiate the involvement of cdk5/p25 in tau hyperphosphorylation in HSV-1-infected neurons. Nuclei were counterstained with Hoechst 33342 (blue). Scale bars: 50 μm.

Cyclin-dependent kinase 5 (Cdk5) is a proline-directed serine/threonine kinase necessary in a wide range of neuronal functions (Kim and Ryan, 2010; Morabito et al., 2004; Meyerson et al., 1992; Malumbres, 2014; Tang et al., 2014) The activity of Cdk5 is restricted to neuronal cells because of the neuron-specific expression of its activators p35 or p39. Aberrant Cdk5 activation caused by the cleavage (mediated by calpain) of p35 to p25 results in tau hyperphosphorylation (Patrick et al., 1999). Considering that calpain can be activated by increased intracellular calcium, which occurs in HSV-1 infected cells (He et al., 2020), we reasoned that the cleavage of p35 and a subsequent formation of Cdk5-p25 complex could account for the hyperphosphorylation of tau in HSV-1 infected neurons. To test this hypothesis, monolayer cultures of neurons were generated from iPSCs following the procedure described in the Methods section. iPSC-derived neurons were infected with HSV-1, strain 17*syn*^+^, at a multiplicity of infection (MOI) of 1. After 24 h, proteins were extracted for subsequent analysis via western blotting analysis. Western blot analysis of HSV-1 infected iPSC-derived neuronal cultures revealed a downregulation of p35 protein levels compared to uninfected controls. Importantly, the cleaved form p25 was not detected in the infected neuronal cultures (Fig. 8 and Supplementary Fig. 5), which indicates that in this neuronal model system an alternative mechanism, independent of cdk5/p25, may contribute to changes in tau phosphorylation upon HSV-1 infection.

#### 2.2.2. Increased Serine-Threonine Kinases BRSK1, BRSK2, TAOK1, and TAOK2 in Infected nuclei

**Fig. 9.**
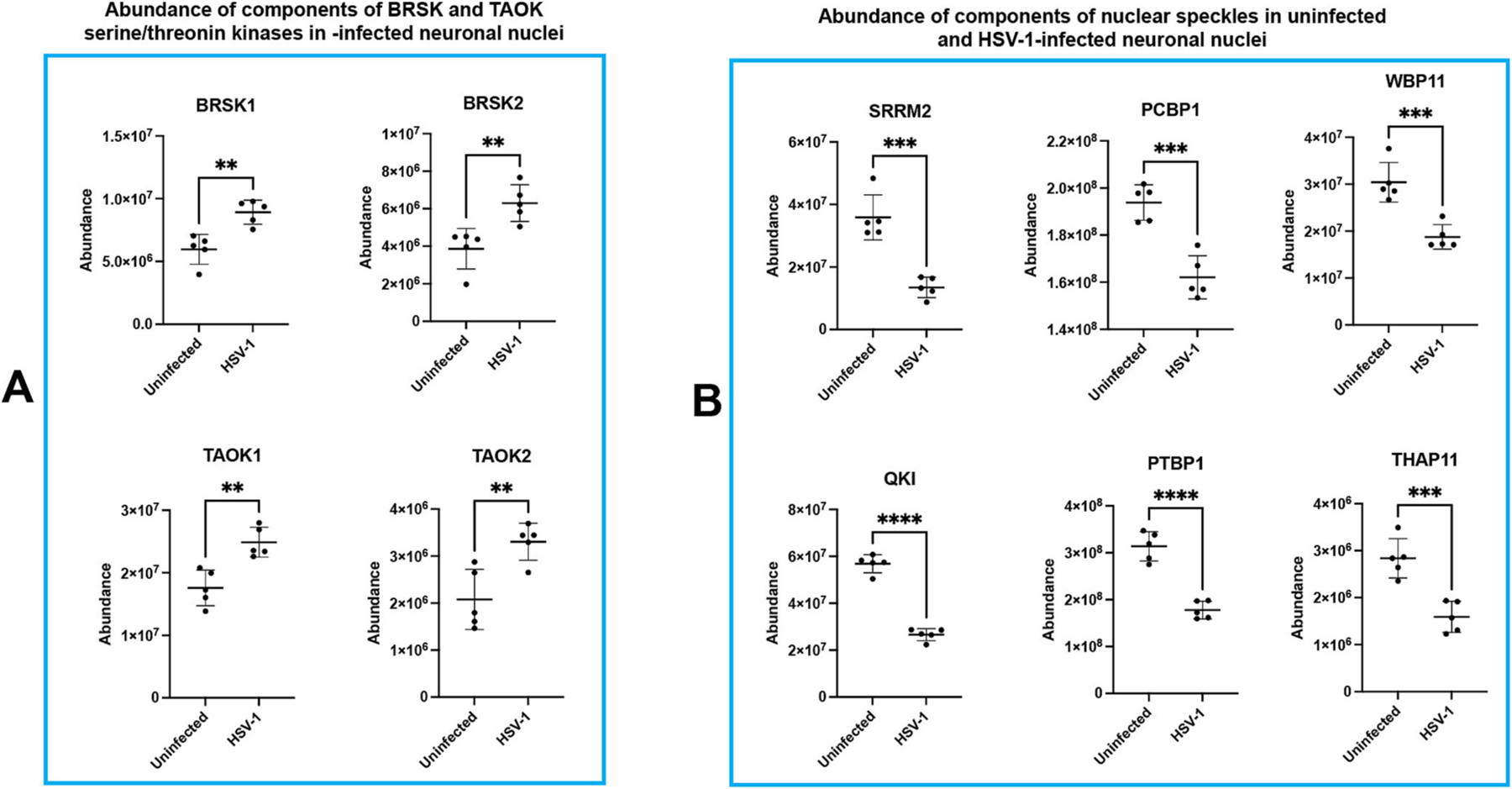
HSV-1 infection causes significant changes in the abundance of nuclear speckle proteins and serine/threonine kinases involved in tau phosphorylation. Mass spectrometry analysis was performed on nuclei isolated from both uninfected iPSC-derived neuronal monolayer cultures and iPSC-derived neuronal monolayer cultures infected with HSV-1 for 24 h, revealing significant changes in the abundance of specific serine/threonine kinases (A) and component of nuclear speckles (B). Notably, significant fold changes were exhibited by SRRM2 (*p* = 0.0002), PCBP1 (*p* = 0.0008), WBP11 (p = 0.0008), QKI (*p* < 0.0001), PTBP1 (p < 0.0001), THAP11 (p = 0.0008), (BRSK1; *p* = 0.0025) and 2 (BRSK2; *p* = 0.0057), as well as thousand-and-one amino acid kinases TAOK1 (*p* = 0.0023) and TAOK2 (*p* = 0.0064). The experiments were performed in quintuplicate. Error bars represent standard deviations. *P* value was determined using Student’s t-test.

During our mass spectrometry analysis of nuclei isolated from uninfected and HSV-1-infected iPSC-derived monolayer neuronal cultures, we unexpectedly observed a significant increase in the abundance of Brain-specific kinases 1 (BRSK1; *p* = 0.0025) and 2 (BRSK2; *p* = 0.0057), as well as thousand-and-one amino acid kinases TAOK1 (*p* = 0.0023) and TAOK2 (*p* = 0.0064) following viral infection (Fig. 9A). These serine/threonine kinases have been shown to phosphorylate tau (Bright et al., 2008; Bendzunas et al., 2024; Fang et al., 2020; Giacomini et al., 2018).

#### 2.2.3. Reduction in the number of nuclear speckles and loss of their globular morphology in low-density host chromatin regions in HSV-1-infected cells

A feature of Alzheimer’s is the dysregulation of mRNA splicing (Raj et al., 2018). Recent research provides evidence of colocalization of nuclear tau aggregates with nuclear speckles, demonstrating alteration in their composition, dynamics, and organization (Lester et al., 2021). The alteration of nuclear speckles, which are crucial for pre-mRNA splicing (Faber et al., 2022), likely contributes to the pathology associated to tau aggregates. Alteration in the organization of nuclear speckles has been described during infections with DNA and RNA viruses (Faber et al., 2022; Chang et al., 2011; Alkalay et al., 2020; Sandri-Goldin et al., 1995; Li and Wang, 2021). Given the link between HSV-1 and AD risk, we investigated the consequence of viral infection on the abundance on nuclear speckle proteins, as well as the number of nuclear speckles and their organization in infected nuclei.

As an exploratory approach to investigate changes in the nuclear protein composition following HSV-1 infection, we employed mass spectrometry (MS) analysis on nuclei isolated from both uninfected and HSV-1 infected monolayer neuronal cultures. IPSC-derived neurons were infected with HSV-1, strain 17*syn*^+^, at MOI of 1. At 24 h post-infection, nuclear extracts were prepared, as described in the Methods section, from uninfected and infected neuronal cultures. An important outcome of the MS analysis was the significant reduction in the abundance with nuclear speckles. The MS analysis revealed a significant reduction in the abundance of nuclear speckle proteins (Galganski et al., 2017) SRRM2 (*p* = 0.0002), PCBP1 (*p* = 0.0008), WBP11 (p = 0.0008), QKI (*p* < 0.0001), PTBP1 (p < 0.0001), THAP11 (p = 0.0008) (Fig. 9B).

**Fig. 10.**
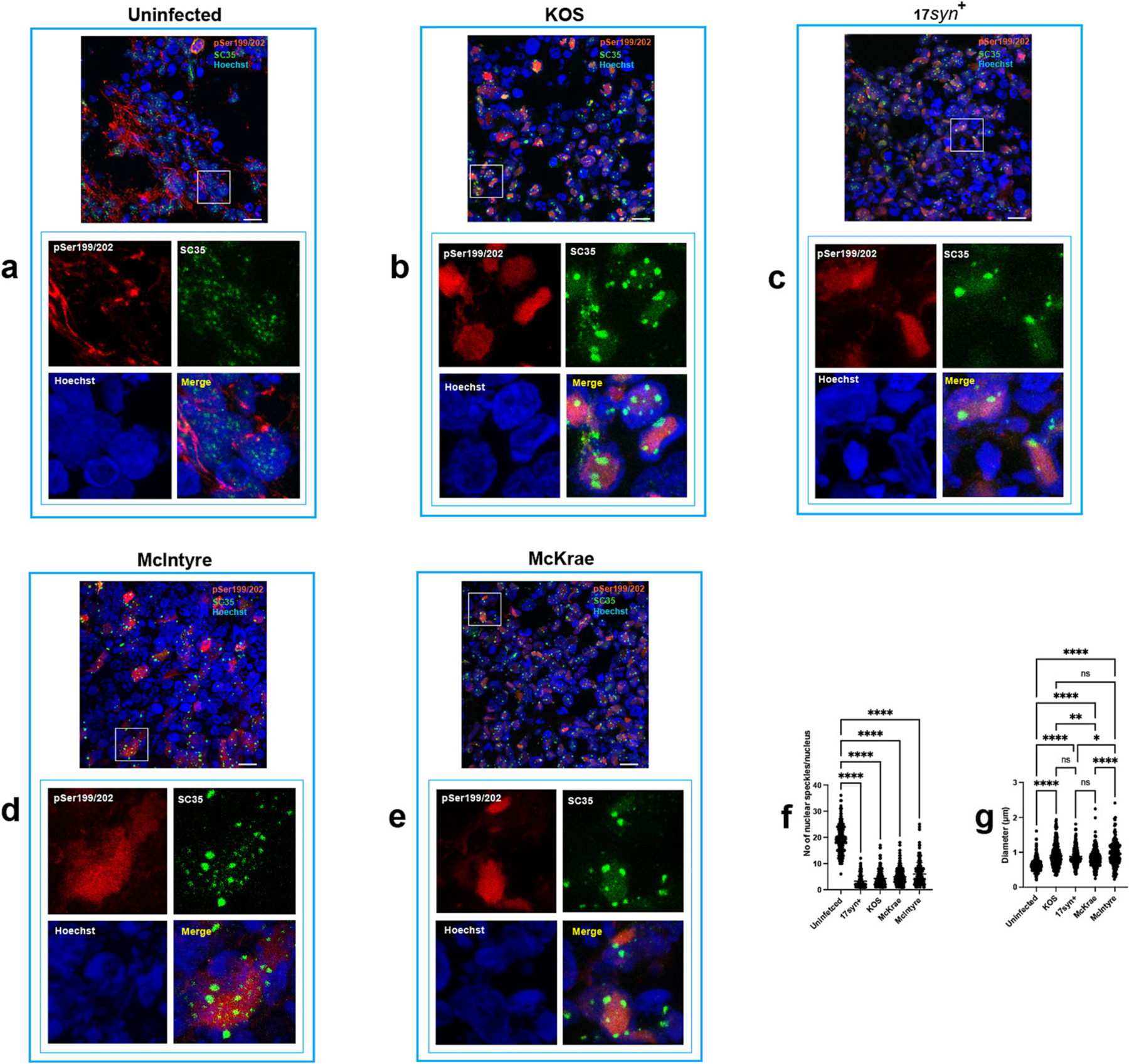
Nuclear speckles undergo to a reduction in number in HSV-1-infected cells and alteration of their compactness in low-density host chromatin regions. Immunohistochemistry analysis was performed on uninfected cortical organoids (A) and organoids infected with HSV-1, strains KOS (B) 17*syn*^+^ (C), McIntyre (D), and McKrae (E) using antibodies against pSer199/202 (red) and the nuclear speckles marker SC35 (green). Each panel displays the co-immunostaining with both antibodies and includes an inset to enhance the visualization of the distribution of nuclear speckles in relation to phosphorylated tau in HSV-1 infected cells. A significant reduction in the number of nuclear speckles (F) and a significant increase in the size of nuclear speckles localized toward the periphery of the low-density host chromatin regions (G) were observed in HSV-1-infected cells. A one-way ANOVA with Dunnet’s multiple comparison test was conducted to assess the reduction in number of nuclear speckles in infect cells. **** *p* < 0.0001. A one-way ANOVA with Dunnet’s multiple comparison test was conducted to assess the increase in size of nuclear speckles localized in infect cells. *p = 0.0165; **p = 0.0085; **** p < 0.0001.

The results of showing a reduction in the abundance of specific nuclear speckle components in 17*syn*^+^-infected cells prompted us to further investigate the impact of various HSV-1 strains with differing neurovirulence (KOS, 17*syn*^+^, McIntyre, and McKrae) on nuclear speckles (Fig. 10). Immunohistochemistry analysis of uninfected (Fig. 10A) and infected organoids (Fig. 10B–E) using an antibody that recognizes the nuclear speckle component SRRM2 (SC35 (Ilik et al., 2020)), showed a reduction in the number of nuclear speckles in infected organoids when compared to the uninfected condition (Fig. 10F). Furthermore, a fraction of nuclear speckles underwent an enlargement in infected cells (Figs. 10B–E, Fig. 10G), when compared to those in the uninfected cells, a phenomenon observed in cells where transcription or splicing are inhibited (Spector et al., 1991; O’Keefe et al., 1994). Enlarged nuclear speckles where mainly localized to the edges of low-density host chromatin regions (Figs. 10B–E). In KOS-infected cells, the speckles located at the periphery of low-density host chromatin regions were larger compared to those in McKrae-infected cells (*p* = 0.0085). Furthermore, the speckles in McIntyre-infected cells were significantly larger than those observed in both 17*syn*^+^- and McKrae-infected cells (*p* = 0.0165 and p < 0.0001, respectively) (Fig. 10G). The impact of HSV-1 on the regulation of gene expression and RNA metabolism was also highlighted by the presence of a diffuse signal corresponding to SC35 within low-density host chromatin regions in a fraction of infected nuclei (Figs. 10B–E), indicating a loss of compactness in nuclear speckles, which was less pronounced in McIntyre-infected cells (Fig. 10D). Co-localization of SC35 and pSer199/202 was observed within low-density host chromatin regions in a fraction of infected cells (Figs. 10B–E). This reveals a complex interplay between HSV-1 infection, changes in the compactness of nuclear speckles, and the nuclear accumulation of phosphorylated tau.

## 3. Discussion

The involvement of HSV-1 in the development of AD remains controversial, with studies showing mixed results regarding the strength of the association and causality between HSV-1 infection and AD pathogenesis (Powell-Doherty et al., 2020; Wozniak et al., 2009a; Brown et al., 2008; Qiao et al., 2022; Wu et al., 2020; McNamara and Murray, 2016; Steel and Eslick, 2015; Wozniak et al., 2009b; Lapeyre et al., 2024; Tran et al., 2021). However, recent studies indicate that neurotropic pathogens may represent important risk factors for neurodegenerative diseases (Blackhurst and Funk, 2023; Levine et al., 2023) rather than a causative agent. The mechanism by which HSV-1 may contribute to tau pathology in neurodegenerative diseases remains unclear. Consequently, investigating the impact of HSV-1 infection on abnormal tau phosphorylation is of great importance and may offer valuable insights into potential avenues for therapeutic strategies. In this study, we employed human induced pluripotent stem cell (iPSC)-derived monolayer neuronal cultures (2D models) and brain organoids (3D models) to model the HSV-1 interaction with human central nervous system. These iPSC-derived neuronal culture systems offer significant advantages over traditional culture systems in that they provide physiologically and disease-relevant models. The physiological relevance and the features of neocortical-like pyramidal neurons make the iPSC-derived 2D and 3D neuronal cultures currently the most relevant in vitro systems for modeling host-pathogen interactions.

The results described in this study show that HSV-1 infection results in an accumulation of phosphorylated tau, corroborating previous findings from Alvarez et al. (Alvarez et al., 2012) Alzheimer’s progression correlates with an increase of nuclear tau phosphorylated at S202/T205 (AT8 antibody epitope) and culminates with the exit of tau from the nucleus (Abasi et al., 2024; Gil et al., 2020; Hernández-Ortega et al., 2016). A discrepancy between our study and Alvarez’s study is that we did not observe accumulation of tau phosphorylated at Ser396, whilst Alvarez et al. showed tau phosphorylated at Ser396/Ser404 in HSV-1 infected cells (Alvarez et al., 2012). This discrepancy could be attributed to differences in the in vitro models employed and the specific phosphorylation sites examined. In their study, Alvarez et al. employed monolayer culture of proliferating SK-N-MC neuroblastoma cells, which were not subjected to neuronal differentiation. Conversely, in our study, we employed differentiated brain organoids, which are mainly composed of cortical neurons. Additionally, the difference between our results and Alvarez’s may be due to different antibodies used. We found no substantial nuclear accumulation using an antibody against tau phosphorylated at S396, while Alvarez et al. used one recognizing tau phosphorylated at both S396 and S404, suggesting tau phosphorylated at S404 accumulates in HSV-1 infected cell nuclei.

The nuclear accumulation of phosphorylated tau in infected cells is associated to the expression of the viral immediate early gene ICP4, which we used as a proxy for HSV-1 viral lytic gene expression (Figs. 3–4). This association was observed, to a lesser extent, during viral reactivation from latency (Fig. 5). Employing chromatin immunoprecipitation (ChIP) analysis using anti-phospho tau pSer199/202 antibody we have demonstrated for the first time a significant enrichment of phosphorylated tau on the HSV-1 chromatin (Fig. 7). Therefore, HSV-1 infection increases phosphorylated tau nuclear accumulation, and at least some of the mobilized phosphorylated tau associates with the viral chromatin. These results lead to the hypothesis that active HSV-1 DNA replication and/or late gene transcription recruits phosphorylated tau to the HSV-1 chromatin. Gaining a better understanding of the reason underlying the accumulation of phosphorylated tau in the nuclei of HSV-1-infected neuronal cells and the details of its interaction with viral chromatin, could pave the way to develop innovative therapeutic strategies for HSV-1-associated neurodegenerative diseases.

Alvarez and coworkers have shown that silencing of tau in neuroblastoma cells using siRNAs had no effect on viral growth (Alvarez et al., 2012). The lack of impact on viral replication when tau is silenced suggests that tau does not exert a direct effect on HSV-1 lytic replication or lytic gene transcription. While Alvarez et al. did not detect an effect of knocking down tau on HSV-1 lytic replication, it is possible that phosphorylated tau’s association with HSV-1 chromatin could help promote the establishment of latency (after the lytic phase). It has been well established that the promotion of heterochromatin formation is a key factor in the suppression of HSV-1 lytic gene transcription as HSV-1 establishes latency (Bloom et al., 2010; Cliffe et al., 2009; Knipe and Cliffe, 2008). Moreover, there are numerous studies linking tau to maintenance of heterochromatin on cellular genes (Mansuroglu et al., 2016; Rico et al., 2021; Maina et al., 2018) and its loss leads to a de-repression of neuronal genes. Therefore, the binding of phosphorylated tau may facilitate repression of HSV-1 lytic genes in neurons during latency and that its loss increases viral protein expression and reactivation.

The presence of nuclear phosphorylated tau in in HSV-1 infected cells was not restricted to neurons but it was also detected in NPCs in the VZ/SVZ regions in the infected brain organoids (Fig. 6). The nuclear accumulation of phosphorylated tau in infected NPCs provides an additional mechanism by which HSV-1 may affect neurogenesis, which is impaired at a pre-symptomatic stage of AD (Mu and Gage, 2011).

In time-course analysis, we observed that phosphorylated tau begins to accumulate in ICP4-positve cells as early as 24 h post-infection (Supplementary Fig. 1). However, it is unclear whether phosphorylated tau translocated from the cytoplasm to the nucleus or if tau phosphorylation occurred in HSV-1-infected nuclei. The staining pattern alone cannot distinguish between these two possibilities. Further studies will be required to establish the cellular compartment where the increase of tau phosphorylation detected in the infected nuclei occurs.

The absence of p35 in HSV-1-infected iPSC-derived neuronal cultures and the lack of detectable expression of p25 (Fig. 8), which is suggested to promote cdk5 hyperactivation (Pao and Tsai, 2021), indicated that an alternative mechanism, independent of cdk5/p25, may contribute to potential changes in tau phosphorylation upon HSV-1 infection of iPSC-derived neurons. Our results differ from previous studies conducted in neuroblastoma cells, where an increase in p35 levels was observed following HSV-1 infection (Haenchen et al., 2010). This discrepancy likely reflects differences between the cellular models used. iPSC-derived neurons represent a more physiologically relevant model of mature, post-mitotic neurons, whereas neuroblastoma cells are immortalized, proliferating cells that may not fully recapitulate the properties of terminally differentiated neurons. This difference in cellular state and maturity likely accounts for the divergent p35/p25 expression patterns observed upon HSV-1 infection. The divergent responses to HSV-1 infection observed in these two models underscore the importance of cellular context in virus-host interactions. P39 is another important activator of CDK5 (Li et al., 2016). However, p39 exerts its function primarily in postnatal neurons, and it exhibits a negligible expression level in pre-natal neurons (Li et al., 2016). Human iPSC-derived neurons appear to correspond to an early developmental stage, resembling fetal or neonatal neurons, rather than mature adult neurons. Importantly, our RNA-sequencing analysis of uninfected and HSV-1 infected iPSC-derived neurons (deposited in NCBI’s Gene Expression Omnibus database (https://www.ncbi.nlm.nih.gov/geo/query/acc.cgi?acc=GSE111656) showed negligible expression of p39 (CDK5R2 gene), with TPM values <1, whilst p35 (CDK5R1 gene) was robustly expressed (150–220 TPM). These evidence highlights the need to reassess the proposed role of CDK5 in tau hyperphosphorylation induced by HSV-1 infection. In fact, it has been previously shown that roscovitine, a cyclin-dependent kinase inhibitor that inhibits Cdk2 and Cdk5, prevents increase of tau phosphorylation and nuclear accumulation of phosphorylated tau (Alvarez et al., 2012). The observation that roscovitine can inhibit tau phosphorylation in HSV-1 infected cells despite the downregulation of p35, the coactivator of CDK5, suggests that CDK2 may be involved or there may be alternative mechanisms participating in tau phosphorylation in infected neurons. There is evidence in the latter hypothesis that roscovitine prevents the initiation of transcription of HSV-1 genes (Diwan et al., 2004) and inhibits effect on HSV-1 replication (Schang et al., 1998).

The mass spectrometry analysis identified several serine/threonine kinases that potentially contribute to tau phosphorylation in infected cells. Fig. 9A demonstrates a significant increase in the abundance of serine/threonine kinases BRSK1, BRSK2, TAOK1, and TAOK2 in HSV-1 infected nuclei. BRSK1 and BRSK2 are known to phosphorylate tau at Ser262 (pS262) (Bendzunas et al., 2024). Furthermore, thousand-and-one amino acid kinases (TAOKs) 1 and 2 have been shown to phosphorylate tau on more than 40 residues (Giacomini et al., 2018). These phosphorylation sites include several associated with AD, such as T123, T427, S262/S356, and S202/T205/S208 (Giacomini et al., 2018; Ikura et al., 1998). A significant finding is that active TAOKs are spatially associated with both pre-tangle and tangle formations in AD brain sections (Giacomini et al., 2018). This spatial correlation suggests a potential role for these kinases in the pathogenesis of AD-related tau aggregation. Notably, TAOKs bind double-stranded RNA and play a crucial role in triggering the antiviral immune response in both flies and humans (Pennemann et al., 2021), leading to hypothesize that TAOKs may not only trigger antiviral immune response but also contribute to tau phosphorylation. It is worth mentioning that HSV-1 encodes serine/threonine protein kinases (UL13 and US3) (Gershburg et al., 2015). However, their potential role in tau phosphorylation, if any, remains to be determined.

A recent study has demonstrated that nuclear tau aggregates alter nuclear speckles composition, leading to alteration of pre-mRNA splicing (Lester et al., 2021). Several reports have shown altered pre-mRNA splicing in brain tissues of AD patients (Wong, 2013; Sato et al., 1999; Glatz et al., 2006). This observation motivated us to investigate changes in the composition of nuclear speckles in HSV-1-infected iPSC-derived neurons. The mass spectrometry analysis of nuclear extracts from HSV-1 (strain 17*syn*^+^)-infected cells unveiled significant reduction in the abundance of several components of nuclear speckles (Fig. 9B), including SRRM2, which is essential for nuclear speckles formation (Xu et al., 2022). Immunohistochemistry analysis have shown an overall reduction of the number of nuclear speckles (Fig. 10G). The nuclear speckles in infected cells were primarily localized to the edges of low-density host chromatin regions (Figs. 10B–F). Interestingly, a diffused signal corresponding to SC35 was observed in low-density host chromatin regions and colocalized with pSer199/202. These findings lead us to theorize a dual role of nuclear speckles in infected nuclei, where nuclear speckles localized at the periphery of low-density host chromatin regions participate in viral export (Chang et al., 2011), whilst those which have undergone a loss of their integrity, exhibiting a diffused pattern in low-density host chromatin regions, contribute to an efficient viral mRNA processing. Results depicted in Fig. 10 indicate that different HSV-1 strains may have varying effects on nuclear speckles. The less pronounced loss of compactness in McIntyre-infected cells might suggest that this strain may have a distinct interaction with nuclear speckles, potentially affecting the gene expression and RNA processing of host cell differently than other strains. Considering the role of nuclear speckles in RNA processing and the regulation of gene expression, a less pronounced alteration in their structure could imply that McIntyre-infected cells maintain more stable gene expression and RNA processing activities.

Considering the effect of nuclear phosphorylated tau on nuclear speckles, it can be hypothesized a mechanism where nuclear phosphorylated tau binds the viral chromatin but concurrently affects the structural integrity of a fraction of nuclear speckles. This loss of interaction between the components of the nuclear speckles and their diffusion in low-density host chromatin regions may be required to enhance viral transcription. Thus, it is conceivable that nuclear phosphorylated tau may serve a dual function by facilitating the expression of specific viral genes and/or contributing to the establishment of latency. These results may signify a potential mechanism through which HSV-1 infection contributes to AD.

In summary, our study analyzes for first time the effect of HSV-1 on nuclear tau phosphorylation and nuclear speckles rearrangement in the context of a physiologically relevant human 3D in vitro model. While previous studies (Alvarez et al., 2012; Faber et al., 2022; Chang et al., 2011; Sandri-Goldin et al., 1995) have reported on aspects of nuclear phosphorylated tau accumulation and nuclear speckle rearrangement in less complex 2D cell models, our work significantly builds on these findings in several important ways: 1) the use of brain organoids allows the investigation of the consequences of HSV-1 infections in a system that more accurately reflects in vivo conditions; 2) it provides a comprehensive analysis of nuclear tau phosphorylation analyzed nuclear tau phosphorylation during acute infection, latency, and reactivation from latency; 3) it provides evidence of the ability of nuclear phosphorylated tau to interact with HSV-1 chromatin; 4) it identifies novel kinases that may be involved in nuclear tau phosphorylation during HSV-1 infections. 5) it shows strain-specific effects on nuclear speckle size, indicating strain-specific difference in the ability to disrupt RNA processing or gene expression. Furthermore, our study reveals a loss of structural organization of a portion of nuclear speckles in infected nuclei.

Collectively, our findings suggest a regulatory role of phosphorylated tau in modulating the viral life cycle and shows that HSV-1 infection triggers cellular mechanisms that are commonly observed in Alzheimer’s disease. It is important to note that the message of our study is not to provide additional evidence which reinforces the association between HSV-1 and Alzheimer’s disease. Rather, our results seek to contribute to the understanding of how viral infections may influence cellular processes relevant to neurodegenerative conditions.

## 4. Materials and methods

### 4.1. Cell lines

Vero cells (CCL-81; ATCC; Manassas, Virginia, United States) for plaque assay were maintained in Dulbecco’s modified Eagle’s medium (DMEM, D6171; Millipore Sigma; St. Louis, Missouri, United States) supplemented with 10 % fetal bovine serum (FBS, HyClone; Millipore Sigma; St. Louis, Missouri, United States) and 5 % antibiotic/antimycotic (Anti/Anti, Gibco 15,240–062; ThermoFisher Scientific; Pittsburgh, Pennsylvania, United States).

Human iPSC lines SC0000019 (subclone SF) SC0000020 and (subclone SD) (RUCDR Infinite Biologis, Piscataway, New Jersey, United States) were employed to generate monolayer cultures of neurons and brain organoids, respectively. The iPSCs were established at the National Institute of Mental Health (NIMH) Center for Collaborative Studies of Mental Disorders-funded Rutgers University Cell and DNA Repository (RUCDR) (http://www.rucdr.org/mental-health). The control steps included analysis of pluripotency markers NANOG, Oct4, TRA60, TRA811, OSX2 and SSEA4. We subsequently conducted karyotyping, array comparative genomic hybridization (aCGH) assays and short tandem repeat (STR) profiling and compared them with donor genomic DNA to evaluate structural changes in genomic DNA during the generation of iPSC lines. Cultures were periodically tested for mycoplasma contamination.

### 4.2. Generation of brain organoids

Organoids were generated as previously described (Abrahamson et al., 2020) with some modifications. Briefly, iPSCs cultured with mTeSRTM plus medium (STEMCELL Technologies) in Matrigel-coated 6-well plates were detached with Accutase and then dissociated into single cell suspension by gently pipetting. Cells were resuspended in mTeSR plus medium supplemented with Rho-associated protein kinase inhibitor (ROCK inhibitor) Y27632 (STEMCELLTM) (10 μM) and FGF-b (20 ng/ml) and seeded into low attachment 96-well plate (Nunclon Sphera 3D culture system, ThermoFisher) at the density 10,000 cells/well to allow for embryoid bodies formation. On day 3, the medium was replaced with Essential-6 medium (Gibco, A1516401) supplemented with dual SMAD inhibitors SB431542 10 μM (MilliporeSigma S4317–5MG) and LDN193189 100 nM (MilliporeSigma SML055–9–25MG). Half medium was changed every other day. On day 8, medium was replaced with Neuronal medium [DMEM/F12 supplemented with 1× MEM Nonessential Amino Acid supplement (MEM-NEAA, CORNINGÒ 25-025-CI), 1× Glutamax (Gibco 35,050–061), 1× N2 supplement (Gibco 17,502–048) and 1 μg/ml Heparin (STEMCELLTM 07980), 20 ng/ml rhFGF-basic, and 20 ng/ml rhEGF] in untreated 10-cm petri dishes (Corning, #430591). Half medium was changed every other day. On day 16, spheres were transferred into 10-cm Petri dishes in Neuronal medium and placed on an orbital shaker in the incubator at 70 rpm (Orbi-BlotterÔ). Medium was changed every 3 days. On day 20, culture medium was changed to cortical organoid differentiation medium I [CODMI: DMEM:F12/Neurobasal (1:1 *v*/v) supplemented with 1× Glutamax, 1× B-27 (VitA[-]), 0.5× Non-essential amino acids, 0.5× N-2, Insulin (2.5 mg) and 1× penicillin-streptomycin (P/S), 20 ng/ml rhFGF-basic, and 20 ng/ml rhEGF]. Five days later, CODM-I medium was replaced with cortical organoid differentiation medium II (CODM-II: DMEM/F12-Neurobasal (1:1 v/v) supplemented with 1× glutamax, 1× B-27 (VitA[+]), 0.5× Non-essential amino acids, 0.5× N-2, Insulin (2.5 mg), BDNF (10 ng/ml), and 1× P/S. Culture medium was changed every other day. On day 42, CODM-II medium was replaced with BrainPhys^™^ Neuronal medium (StemCell Technologies).

### 4.3. Virus preparation

The virus stocks were prepared in the D’Aiuto laboratory at the University of Pittsburgh. 80–90 % confluent monolayers of Vero cells were infected at a multiplicity of infection (MOI of 3 in DMEM medium supplemented with 2 % FBS. After 2 h the inoculum was removed, cells were washed and cultured for 2–3 days, until the appearance of full cytopathic effect (CPE). The cells were scraped and transferred along with the culture supernatant into 15 ml conical tubes. Cells were centrifuged at 1000 rpm for 5 min. The culture supernatant was removed, leaving behind 1.5 ml, and the cell pellet was resuspended using a vortex for 1–2 min. Cells were freeze-thawed three times. Debris was then removed by centrifuging at 3000 rpm for 5 min and the top culture supernatant containing cell-free viral particles was stored at −80 °C until use. Virus titers were determined by standard plaque assay as described below.

### 4.4. HSV-1 plaque assay

Plaque assays were performed after confluent monolayers of Vero cells in 24-well tissue culture dishes were achieved. Once confluent, cells were infected with serial dilutions of HSV. After infection, supernatants were aspirated, cells were washed with PBS, and one milliliter of 3 % (*w*/*v*) carboxymethyl cellulose (CMC) solution overlay medium was added. Plates were incubated under standard conditions (5 % CO2, 37 °C, and 100 % humidity) for 72 h. The CMC medium was then removed, cells were washed with PBS, and finally fixed in 4 % formalin solution. After one hour, the fixative was removed, and plaques were visualized by staining with gentian violet.

### 4.5. Infection of brain organoids

The following HSV-1 strains were employed in this study: KOS (VR-1493; ATCC); 17*syn*+, McIntyre, and McKrae (gifts from Dr. D. Bloom from stocks obtained from Dr. J. Stevens).

Fifty-one-day-old brain organoids were infected individually in U-bottom low attachment 96-well plates with an HSV-1, strains KOS and 17*syn*^+^ (which exhibit differences in virulence properties) (3000 pfu/organoid). After 2 h, the inocula were removed, the organoids were washed and cultured in BrainPhys^™^ Neuronal medium (StemCell Technologies).

To establish latency, organoids were pre-treated with of the antivirals (*E*)-5-(2-bromovinyl)-2′-deoxyuridine (5BVdU, 30 μM) and interferon-a (IFN-a, 125 U/ml) for 24 h. Two hours after the infection, the inoculum was removed, the organoids washed twice with 500 μl of DMEM-F12 medium and cultured for 7 days in the aforementioned supplemented BrainPhys^™^ Neuronal medium in the presence of 5BVdU + IFN-a. Medium was changed every other day.

To induce viral reactivation, latently infected organoids were treated with phosphatidylinositol 3-kinase inhibitor LY294002 (PI3Ki, 20 mM) for 3 days.

For each independent experimental repeat (*N* = 2 for SC0000019 organoids and N = 2 for SC0000020 organoids), 6–10 organoids were employed per condition.

### 4.6. Generation of monolayer cultures of iPSC-derived neurons

Neuronal precursor cells (NPCs) were derived from iPSCs as follows. iPSCs were seeded at the density of 10^6^ cells/well in matrigel-coated 6-well plate. Cells were cultured in mTeSR1 supplemented with dual SMAD inhibitors SB431542 10 μM and LDN193189 100 nM, and rock inhibitor (10 μM) for 5–7 days until the appearance of neural rosettes. Rock inhibitor was withdrawn after 24 h. Cells were then dissociated with Accutase^™^ (StemCell Technologies) and seeded at the density of 10^6^ cells/well in matrigel-coated 6-well and cultured in mTeSR1 Plus supplemented with dual SMAD inhibitors and rock inhibitor. After 5–7 days NPCs were dissociated and expanded in StemDiff^™^ Neural progenitor medium (StemCell Technologies) in Matrigel coated 6-well plates (10^6^ cells/well).

NPCs were seeded into Matrigel-coated 12- or 6-well plates to the density of 2.5 × 10^5^ or 5 × 10^5^ cells/well, respectively, and cultured in neurobasal medium [Neurobasal medium supplemented with 0.5× B27 (Vitamin A+), 1× penicillin-streptomycin (P/S), 1× Glutamax, BDNF (10 ng/ml), CHIR99021 (3 uM), Dorsomorphin (1 uM), Forskolin, and ROCK inhibitor (10 μM)]. Two days later, CHIR99021, Dorsomorphin, Forskolin (10 uM), and ROCK inhibitor were withdrawn and differentiating NPCs were cultured for 4 weeks. Half medium was changed every other day.

### 4.7. Mass spectrometry

Four-week old iPSC-derived neurons were infected with HSV-1, strain 17syn+, at MOI of 1. After 1 h, the inocula were removed, cells were washed with DMEM/F12 and cultured in neurobasal medium. Cells were harvested after 24 h, and nuclear extracts were prepared using NE-PER^™^ Nuclear and Cytoplasmic Extraction kit (Fisher Scientific) according to manufacturer’s instructions. The nuclei and cytoplasm were brought to a final concentration of 5 % SDS, 50 mM TEAB buffer, probe sonicated, vortexed for 10 min, and centrifuged at 13000 *g* for 10 min at room temperature. Total protein concentration was measured by micro-bicinchoninic acid assay (Thermo Scientific #23235). Protein digestion was carried out on 10 μg of protein from each sample according to the S-trap (Protifi) protocol. Samples were subject to reduction with 20 mM dithiothreitol, heated to 95 °C for 10 min, cooled to room temperature, and alkylated by incubation with 40 mM iodoacetamide in darkness for 30 min at room temperature. Samples were centrifuged at 13000 *g* for 8 min, acidified with 12 % phosphoric acid at 1:10 concentration, and diluted six-fold in binding buffer containing 90 % methanol and 100 mM TEAB at pH 7.1. Samples were dispensed onto S-trap columns which were washed with binding buffer and centrifuged at 4000 *g* for 1 min. Columns were washed with binding buffer, centrifuged at 4000 *g* for 1 min, and transferred to clean tubes for incubation at 47 °C in 50 mM TEAB for trypsin digestion at 1:10 enzyme/substrate. Samples were eluted with a series of solutions including 50 mM TEAB, 0.2 % formic acid, and 50 % acetonitrile with 0.2 % formic acid, and underwent centrifugation at 1000 *g* for 1 min after each eluent was added. Samples were then dried in a speedvac and resuspended in a solution of 3 % acetonitrile and 0.1 % TFA and desalted using Pierce Peptide Desalting Spin Columns (Thermo Scientific # 89851). Eluants were dried in a speedvac and resuspended in a solution of 3 % acetonitrile and 0.1 % formic acid to a final concentration of 0.5 μg/μL.

Mass spectrometry analysis was conducted on a Thermo Fisher QE-HFX coupled to an Easy-nLC 1200. Approximately 1 μg of each sample was loaded onto an EASY-Spray PepMap RSLC C18 column (2 μm, 100 A, 75 μm × 50 cm) and eluted at 300 nl/min over a 60-min gradient. MS1 spectra were collected at 60,000 resolution with a full scan range of 350–1400 *m*/*z*, a maximum injection time of 50 ms and the automatic gain control (AGC) set to 3e6. The precursor selection window was 1.4 m/z and fragmentation was carried out with HCD at 28 % NCE. MS2 were collected with a resolution of 30,000, a maximum injection time of 50 ms and the AGC set to 1e5 and the dynamic exclusion time set to 90s.

Raw MS files were processed in Proteome Discoverer version 2.4 (Thermo Fisher). MS spectra were searched against the human Uniprot/SwissProt database. Peptide and Protein identification were filtered at an FDR of 0.01. The data were deposited in PRIDE (ID: PXD047281).

Genewise abundances were assessed, and differences between the infected and uninfected groups were analyzed using two-tailed Student’s *t*-test (df = 8). FDR corrected *p*-values were used to assess experiment-wide significance.

### 4.8. Western blotting

Four-week old iPSC-derived neurons were infected with HSV-1, strain 17syn+, at MOI of 1. After 1 h, the inocula were removed, cells were washed with DMEM/F12 and cultured in neurobasal medium. Cells were harvested after 24 h and cell lysates were prepared from uninfected and infected cells with 10 volumes of RIPA Lysis and Extraction Buffer (ThermoFisher). Protein concentration was determined by BCA assay (ThermoFisher). Twenty micrograms of protein were used for western blot by adding 4× Laemmli buffer and same lysis buffer to keep same volume of all samples. Samples were heated at 95 °C for 5 min. Cell lysates were separated by electrophoresis on 10 % SDS-polyacrylamide gels, transferred onto nitrocellulose membranes (Bio-Rad) and probed with rabbit monoclonal antibody to p35/25 (Cell Signaling, C64B10), mouse monoclonal anti-CDK5 antibody (Thermo-Fisher, DC34) and mouse monoclonal anti-ICP4 antibody (Abcam, ab6514).

In a parallel experiment, nuclei from uninfected and infected iPSC-derived neurons underwent lysis using RIPA buffer. Protein samples were prepared as described above and separated using a 4–12 % SDS-PAGE gel (Invitrogen), then transferred to a nitrocellulose membrane (Li-COR #926–31,092). After a blocking step, anti-Tau phospho Serine 199/202 (Millipore-Sigma Cat#AB9674 (dilution 1:500) and anti-lamin (Abcam # ab16048; 1:500) antibodies were added to the membrane and incubated simultaneously at 4 °C overnight. Following washing and detection of bound antibody using nIR fluorophore-conjugated secondary antibodies (IRD680 goat anti-mouse, 1:10,000, LI-COR, 926–68,070; IRD800 goat anti-rabbit, 1:10,000, Li-COR, #926–32,211) the blot was imaged using a Li-COR Odyssey scanner.

### 4.9. Immunohistochemistry

Frozen sections of organoids were prepared as follows. Organoids were transferred into cryomolds (Tissue-Tek Cryo Mold Intermediate). After adsorbing traces of culture medium, the organoids were embedded into OCT medium in cryomolds (Tissue-Tek Cryo Mold Intermediate) and frozen at −22 °C. 10-μm sections were prepared by Cryostat (Micron HM350; Thermo Fisher Scientific). From each condition, a minimum of 30 sections were obtained by cryosectioning the organoids. Each cryosection contained 2–3 organoids. At least 3 slides from each condition have been analyzed. To prevent sampling the same cells only sections that were 50 μm apart were used. Frozen sections were stored at −80 °C until needed. Before staining, frozen sections fixed with 4 % paraformaldehyde for 20 min, and incubated with Goat serum (Thermo Fisher Scientific, # 50062Z) and Triton-X 100 (Millipore Sigma, #X-100) for 1 h at room temperature.

Samples were incubated with primary antibodies overnight at 4 °C. Primary antibodies used were mouse monoclonal anti-HSV-1 ICP4 (Abcam Cat# ab6514, dilution 1:500), rabbit polyclonal anti-Tau phospho Serine 199/202 (Millipore-Sigma Cat#AB9674, dilution 1:250), rabbit polyclonal anti-Tau phospho Threonine 205 (Invitrogen Cat# 44–738G, dilution 1:250) rabbit polyclonal anti-Tau phospho Serine 396 (Invitrogen Cat# 44–752G, dilution 1:250), mouse monoclonal anti-HSV-1 ICP27 (Virusys, Cat# P1113, dilution 1:500), and mouse monoclonal anti-SC35 (Abcam, Cat# ab11826, dilution 1:250).

The following fluorophore-conjugated secondary antibodies were used to detect bound primary antibodies: Alexa Fluor 488 goat anti-rabbit (Thermo Fisher Scientific Cat# 1:300 dilution), Alexa Fluor 488 goat anti-mouse (Thermo Fisher Scientific Cat# A-10680, 1:300 dilution), Alexa Fluor 594 goat anti-rabbit (Thermo Fisher Scientific Cat# A-11012, 1:300 dilution), Alexa Fluor 594 goat anti-mouse secondary antibody (Thermo Fisher Scientific Cat# A-11005, 1:300 dilution). A fluorescent microscope (Leica CTR5500) was used for image acquisition. Also, images were acquired with a Nikon A1 confocal with a 60× oil objective. Z Stacks were acquired at 0.5 μm steps, 1024 × 1024 resolution, using conservative laser power settings.

Pearson’s R coefficient and Manders’ overlap coefficient to quantify colocalization between ICP4 and pSer199/202 were calculated using JaCoP plug-in feature in Fiji. A threshold of *R* > 0.5 was set to define high colocalization between the two markers, as this value is generally considered to represent a strong positive correlation.

The fluorescence intensity of nuclear pSer199/202 in uninfected and infected organoids was analyzed by determining the corrected total cell fluorescence (CTCF) using Fiji and normalizing for background using the equation: CTCF = Integrated Density - (Area of selected cell x Mean fluorescence of background readings).

Nuclear speckles were analyzed in both uninfected and HSV-1 infected nuclei using a combination of automated image analysis and manual counting. Initial image analyses were performed using Fiji software, with initial first pass analysis employing auto-thresholding. The counts obtained from automated analyses were validated by performing manual counts in a masked fashion. An unacceptable level of discrepancies occurred between automated and manual counts and were attributed to several factors, including disaggregation of a fraction of nuclear speckles in infected nuclei and difficulties of the software identifying speckles when located in certain regions of the nucleus. Therefore, to ensure the accuracy of our data, we conducted manual counting of nuclear speckles in all images, applying consistent criteria across all samples and in a masked fashion.

The diameters of the nuclear speckles at the edge of low-density host chromatin regions were measured individually using Fiji.

#### 4.9.1. RNAscope^®^ HiPlex in situ hybridization

Frozen organoid sections were processed for RNA in situ transcript analysis at the indicated days post-infection along with age-matched Mock infected sections following protocols developed by ACD for fixation and dehydration. Organoid sections were removed from −80 °C and immediately immersed in a 4 % formaldehyde/PBS solution for 15 min at room temperature followed by dehydration in an ethanol series as follows: 50 % for 5 min, 70 % for 5 min, 100 % for two times at 5 min each, and sections allowed to air dry for 5 min at room temperature. Two rounds of in situ hybridization were performed using probes, reagents, and protocols designed by ACD to detect four different RNA transcripts in each cell through their proprietary HiPlex assay system. We performed HiPlex according to ACD’s recommendations. Briefly, organoid sections were digested with Protease III (ACD) for 30 min at 40 °C, rinsed two times with distilled water, and incubated with ACD-designed probes against HSV-1 transcripts LAT, ICP4, VP16 and gC. For RNA hybridization, stock 50× concentrated probes were diluted to 1× in ACD probe dilution buffer and incubated with organoid sections for 2 h at 40 °C. Following hybridization, sections were washed two times for 2 min each with ACD RNAscope wash buffer. ACD probes were amplified following the manufacturer’s recommendation followed by incubation with the appropriate HiPlex fluorophores for 15 min at 40 °C. Sections were washed two times for 2 min each with ACD RNAscope wash buffer, stained with DAPI, and cover slipped with Pro-Long gold mounting media (ThermoFisher Sci., catalog no. P10144). Imaging was performed using a Nikon Eclipse E600 microscope with epifluorescence and sections photographed with a Qimaging Exi Aqua Monochrome Digital camera. For second round detection of RNA transcripts, cover slips were soaked off overnight by submersion in 4× SSC followed by cleavage of the fluorophores using reagents and protocols developed by ACD. Sections were then incubated with the appropriate HiPlex fluorophores for 15 min at 40 °C and processed for microscopy as described for first round transcript detection.

### 4.10. Chromatin Immunoprecipitation (ChIP) qPCR

IPSC-derived neuronal cultures were infected with HSV-1, strain KOS and a multiplicity of infection (MOI) of 1. After 24 h, infected cells (approx. 3 × 10^6^) were harvested for ChIP by fixation with formaldehyde/PBS (1 % final) for 8 min at room temperature, quenched with glycine (0.125 M final) for 5 min at room temperature, and cells washed two times with ice cold PBS. Cell pellets were resuspended in 1 ml RIPA buffer (10 mM Tris-HCL pH 8.0, 1 mM EDTA, 140 mM NaCl, 0.5 % sodium dodecyl sulfate, 1 % Triton X-100, 0.1 % sodium deoxycholate) plus protease inhibitors (Thermo Fisher Scientific, Halt Protease Inhibitor Cocktail, catalog no. 1862209), incubated on ice for 10 min and sonicated for 12 cycles at 30s on / 30s off (Diagenode, Bioruptor^®^). Lysates were clarified by centrifugation at 13,000 rpm for 10 min at 4 °C. Five percent of chromatin was saved for calculation of input. The remaining chromatin was diluted five-fold with ChIP dilution buffer (10 mM Tris-HCL pH 8.0, 1 mM EDTA, 140 mM NaCl, 0.1 % SDS, 1 % Triton, 0.1 % sodium deoxycholate) containing protease inhibitors and incubated with (1) polyclonal anti-Tau phospho Serine 199/202 (Millipore-Sigma Cat#AB9674) and (2) rabbit IgG overnight at 4 °C with rotation. The following day, 20 μl ChIP-Grade Protein A/G Magnetic Beads (Thermo Fisher Scientific, catalog no. 26162) were added to each sample and incubated at 4 °C with rotation for 4 h. The beads were washed three times for 5 min each with ChIP dilution buffer and then one time for 5 min with high salt buffer (20 mM Tris-HCL pH 8.0, 500 mM NaCl, 2 mM EDTA, 1 % Triton X-100, 0.1 % sodium dodecyl sulfate) with rotation. Chromatin was eluted from the beads two times by incubating with 75 μl Elution Buffer each (50 mM Tris-HCL pH 8.0, 1 mM EDTA, 1 % sodium dodecyl sulfate) at 65 °C for 30 min with vortexing every 10 min and the elutions combined. ChIPed chromatin and input samples were de-crosslinked by incubation with 10 μl 5 M NaCl at 95 °C for 5 min followed by RNAse A (Thermo Fisher Scientific, catalog no. EN0531) treatment for 30 min at 37 °C and then Proteinase K (Sigma, catalog no. P4850) digestion at 65 °C overnight. Chromatin was purified for qPCR using columns following the manufacturer’s recommendation (Zymo Research, ChIP DNA Clean & Concentrator, catalog no. D5205). qPCR was performed on a Roche LightCycler^®^ 480 II thermocycler using TaqMan fast universal PCR master mix (Thermo Fisher Scientific/Applied BioSystems, catalog no. 4352042) and custom primer probes. Standard curves were prepared by serial dilution of the input sample and amount of chromatin immunoprecipitated was calculated by the following equations: DCt [normalized Chip] = (Ct [ChIP] - (Ct [Input] - Log2 (Input Dilution Factor) and then Input % = 100/2^DCt [normalized ChIP]^. Primers spanning the HSV-1 genome were used for qPCR:

#### LAT pro:

F (5’ CAATAACAACCCCAACGGAAAGC 3′), R (5’ TCCACTTCCCGT CCTTCCAT 3′),

Probe: (5’ TCCCCTCGGTTGTTCC 3′);

#### ICP4:

F (5’ CACGGGCCGCTTCAC 3′), R (5’ GCGATAGCGCGCGTAGA 3′),

Probe (5’ CCGACGCGACCTCC 3′);

#### ICP0 pro:

F (5’ CCGCCGACGCAACAG 3′), R (5’ GTTCCGGGTATGGTAATGAGTTTCT 3′).

Probe (5’ CTTCCCGCCTTCCC 3′);

#### TK:

F (5’ CACGCTACTGCGGGTTTATATAGAC 3′), R (5’ GGCTCGGGT ACGTAGACGATAT 3′), Probe (5’ CACCACGCAACTGC 3′);

#### POL:

F: (5’ AGAGGGACATCCAGGACTTTGT 3′), R (5’ CAGGCGCTTG TTGGTGTAC 3′).

Probe (5’ ACCGCCGAACTGAGCA 3′);

#### VP16:

F (5’ CTTGGTCGACGAGCTGTTTG 3′), R (5’ GCCCCGTTGCGTACAG 3′),

Probe (5’ CCGTCCGCGTTCATG 3′);

#### gC:

F (5’ CCTCCACGCCCAAAAGC 3′), R (5’ GGTGGTGTTGTTCTTG GGTTTG 3′),

**Probe (5’ CCCCACGTCCACCCC 3**′**).**

#### Antibodies for ChIP:

Rabbit Anti-Tau phosphoserine 199/202 polyclonal antibody (EMD Millipore Corp., catalog no. AB9674, and Rabbit IgG, polyclonal - Isotype Control ChIP Grade (Abcam, catalog no. ab171870).

## Supplementary Material

1

2

3

4

5

6

## Data Availability

The datasets generated and/or analyzed during the current study are available in the PRIDE (ID: PXD047281), https://www.ebi.ac.uk/pride/.
