# Supplementary figures and images for: Phosphorylated-tau associates with HSV-1 chromatin and correlates with nuclear speckles decondensation in low-density host chromatin regions
Source: Neurobiol Dis. Author manuscript; Available in PMC 2025 Apr 16. (PMC12001802; doi:10.1016/j.nbd.2025.106804)

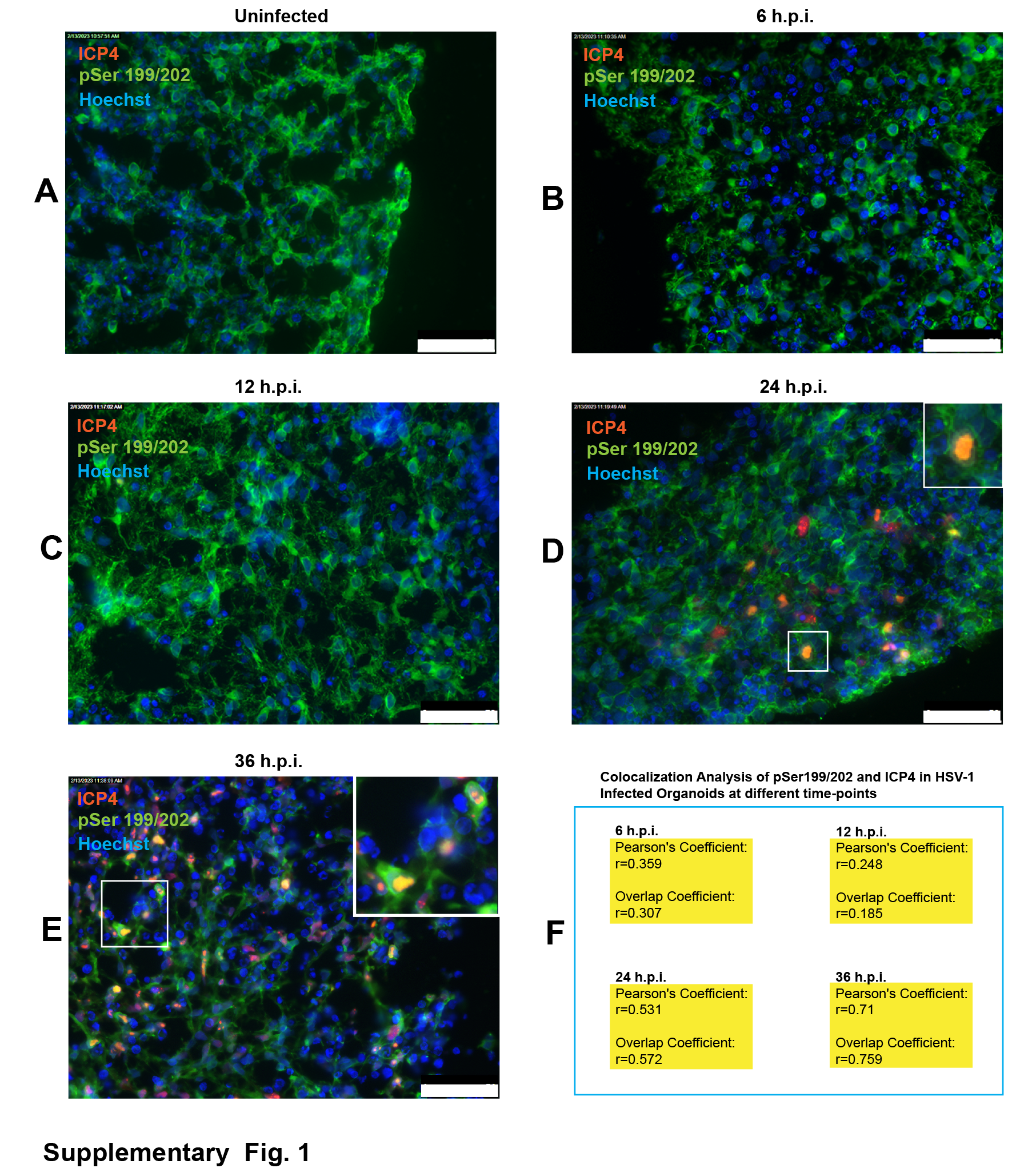

Supplement: 1 [file NIHMS2060007-supplement-1.zip › mmc1.tif]

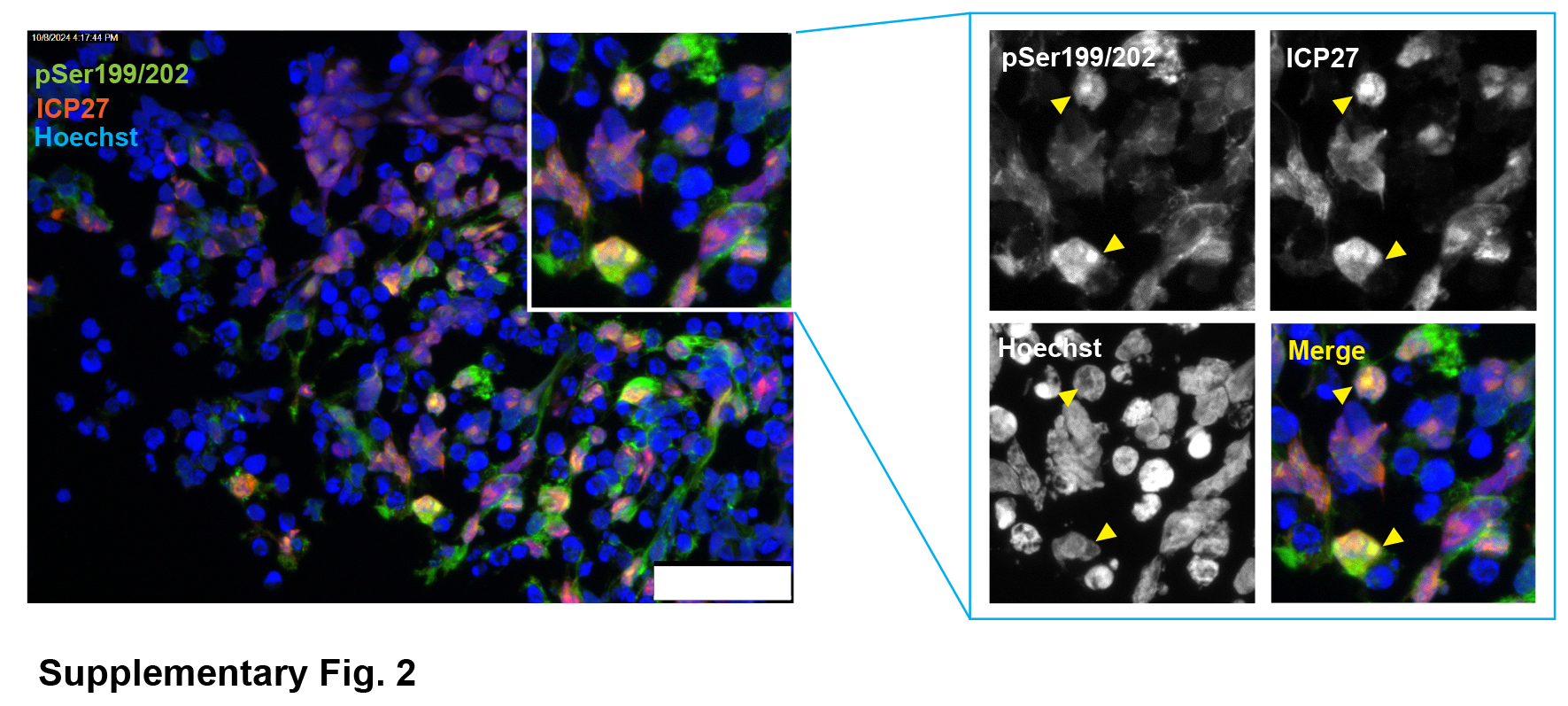

Supplement: 2 [file NIHMS2060007-supplement-2.zip › mmc2.tif]

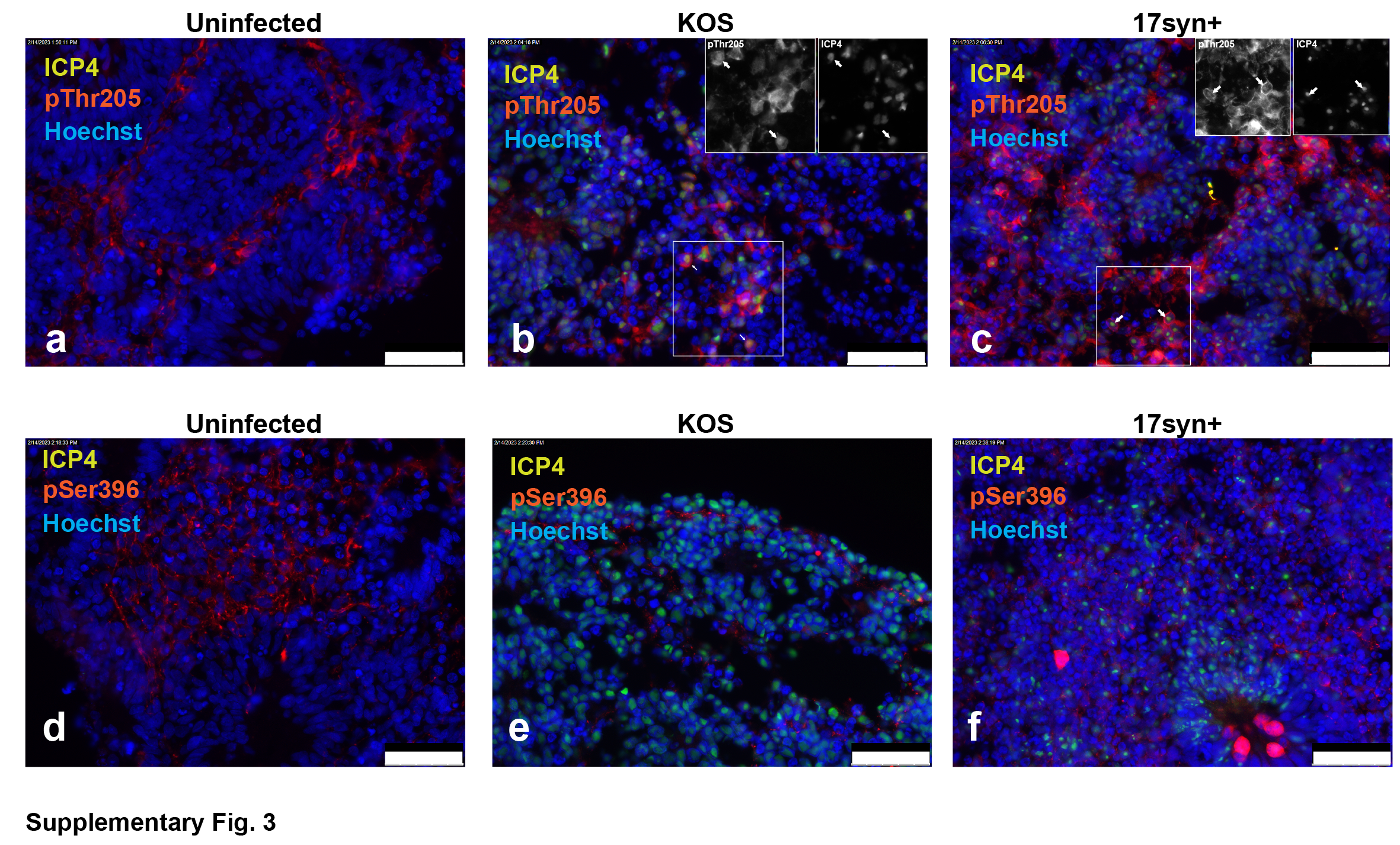

Supplement: 3 [file NIHMS2060007-supplement-3.zip › mmc3.tif]

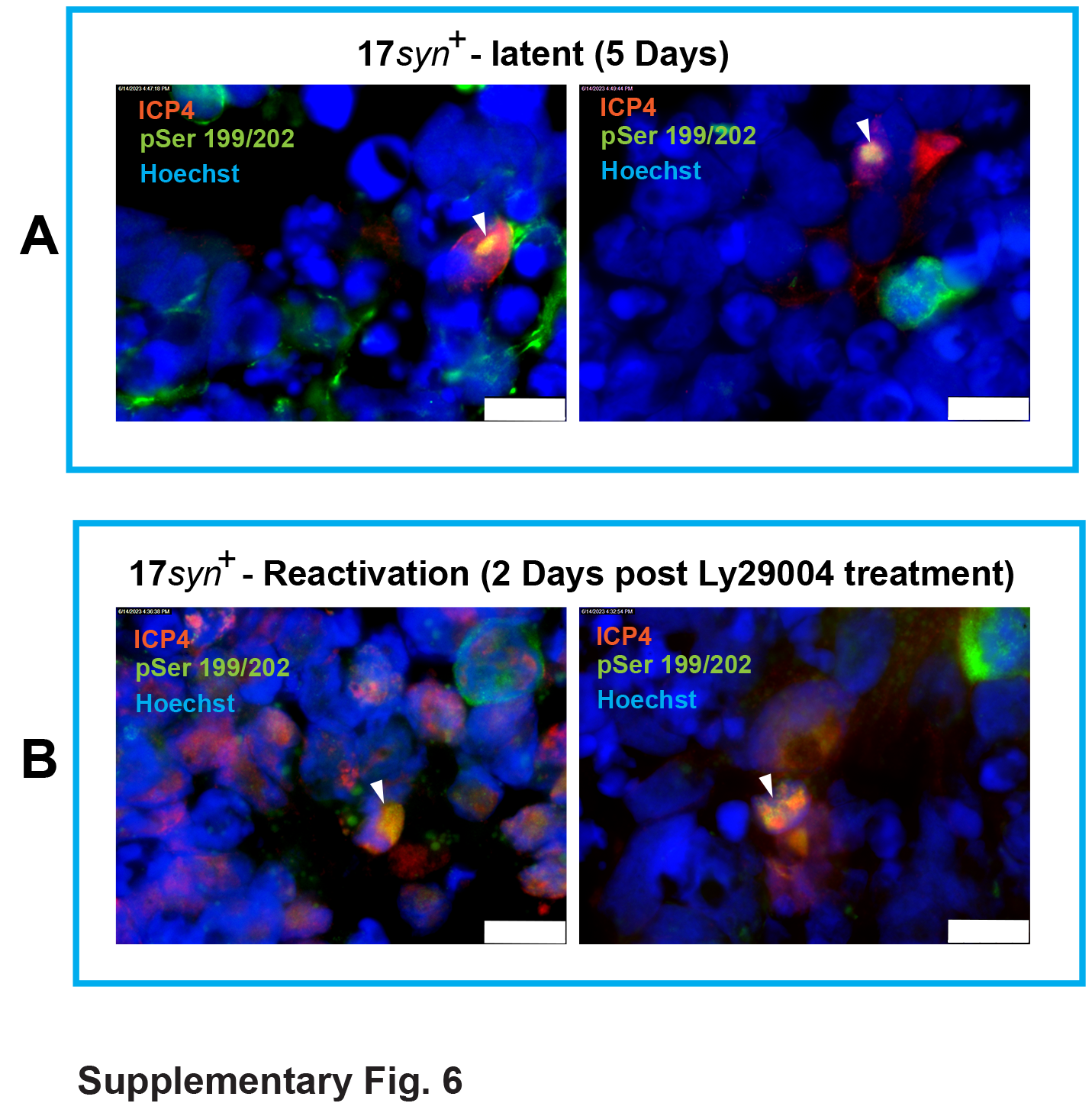

Supplement: 4 [file NIHMS2060007-supplement-4.zip › mmc4.tif]

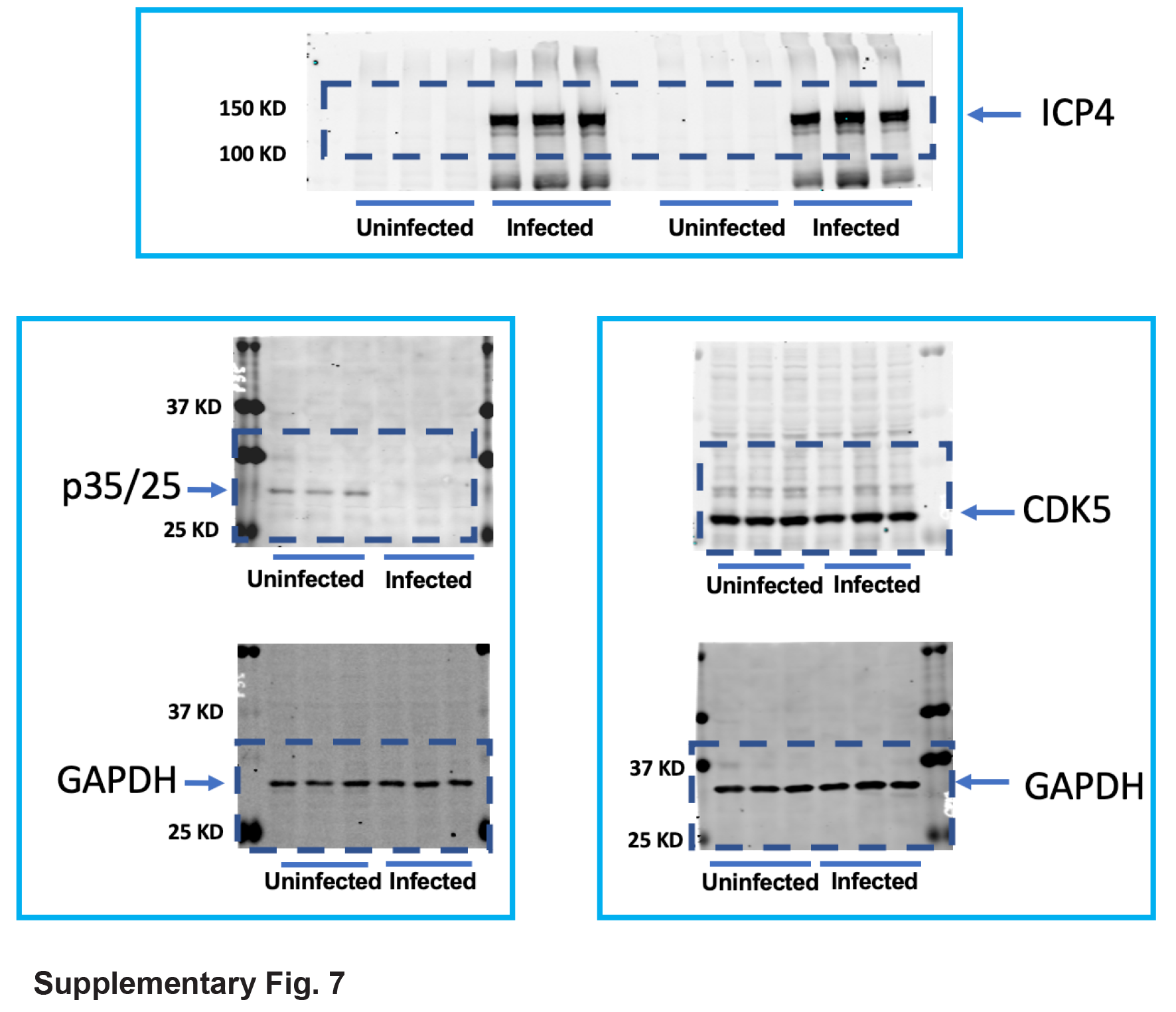

Supplement: 5 [file NIHMS2060007-supplement-5.zip › mmc5.tif]
