# Supplementary material for: Phosphorylated-tau associates with HSV-1 chromatin and correlates with nuclear speckles decondensation in low-density host chromatin regions
Source: Neurobiol Dis. Author manuscript; Available in PMC 2025 Apr 16. (PMC12001802; doi:10.1016/j.nbd.2025.106804)
Supplement: 6 [file NIHMS2060007-supplement-6.docx]

**Supplementary Figure 1.** **Time course analysis of phosphorylated tau in ICP4-Positive cells during acute infection of cortical organoids.** Organoids were infected individually with HSV- 1, strain KOS (3000 pfu/organoid). After 2 hours the inocula were removed and organoids were processed immunohistochemistry at different time points. Representative images show pSer199/202 (green) and ICP4 (red) stainings in (**A**) uninfected organoids and infected organoids at 6 (**B**), 12 (**C**), 24 (**D**), and 36 (**E**) hours post infection (h.p.i.). F) Quantitative Colocalization Analysis of pSer199/202 and ICP4 in HSV-1 infected organoids. Pearson’s R coefficient and Manders’ overlap coefficient were calculated using JaCoP plug-in feature in Fiji. Nuclei were counterstained with Hoechst 33342. Scale bar: 50 μm.

**Supplementary Figure 2.** Co-localization of nuclear tau phosphorylated at pSer199/202 with HSV-1 immediate early gene ICP27 in low-density host chromatin regions. Examples of co-localizations of the viral protein ICP27 (red) with nuclear phosphorylated tau (pSer199/202) (green) are indicated by arrowheads. Nuclei were counterstained with Hoechst 33342 (blue). Scale bar, 50 μm.

**Supplementary Figure 3.** Tau phosphorylated at Thr205, but not at Ser396 is detected in the nuclei of HSV-1 infected cells. The immunohistochemistry analysis showed nuclear accumulation of phosphorylated tau specifically at Thr205 (red) and its colocalization with ICP4 (green) in 17*syn*^+^- and KOS-infected organoids (**B**, **C**) at day 3 post-infection. However, conclusive detection of an increase in nuclear Ser396 phosphorylated tau in the infected cells was not observed (**E**-**F**). The immunostaining with tau phosphorylated at Thr205 and Ser396 in uninfected organoids is depicted in (**A**) and (**D**), respectively. Nuclei were counterstained with Hoechst 33342 (blue). Scale bars, 50 μm.

**Supplementary Figure 4.** Nuclear accumulation of tau phosphorylated at Ser199/202 (green) and its colocalization with ICP4 (red) in 17*syn*^+^-infected organoids, both in latently infected cells expressing ICP4 (indicating delayed or inefficient establishment of latency) (**A**) and following viral reactivation from latency (**B**). Arrowheads indicate co-localization of HSV-1 protein ICP4 with phosphorylated tau in infected organoids. Nuclei were counterstained with Hoechst 33342 (blue). Scale bars, 10 μm.

**Supplementary Figure 5.** Full-length western blot images. Full-length, unedited Western blot images corresponding to the cropped blots presented in the main manuscript in Figure 8. The molecular weight markers are indicated on the left side of each blot. Cropping of the original blot

images is indicated with blue dashed rectangles.
